# Discovery of Mcl-1 inhibitors from integrated high throughput and virtual screening

**DOI:** 10.1038/s41598-018-27899-9

**Published:** 2018-07-05

**Authors:** Ahmed S. A. Mady, Chenzhong Liao, Naval Bajwa, Karson J. Kump, Fardokht A. Abulwerdi, Katherine L. Lev, Lei Miao, Sierrah M. Grigsby, Andrej Perdih, Jeanne A. Stuckey, Yuhong Du, Haian Fu, Zaneta Nikolovska-Coleska

**Affiliations:** 1 Department of Pathology, University of Michigan Medical School, Ann Arbor, MI USA; 2 Molecular and Cellular Pathology Graduate Program, University of Michigan Medical School, Ann Arbor, MI USA; 3 Interdepartmental Graduate Program in Medicinal Chemistry, University of Michigan, College of Pharmacy, Ann Arbor, MI USA; 4 Program in Chemical Biology, University of Michigan, Ann Arbor, MI USA; 5 National Institute of Chemistry, Ljubljana, Slovenia; 6 Life Sciences Institute, University of Michigan, Ann Arbor, MI USA; 7 Department of Pharmacology, Emory University, Atlanta, GA USA; 8 Present Address: School of Medical Engineering, Hefei University of Technology, Hefei, Anhui 230009 China; 9 Present Address: Pfizer Inc, Lake Forest, IL 60045 USA; 10 Present Address: Basic Research Laboratory, National Cancer Institute, Frederick, MD 21702 USA

## Abstract

Protein-protein interactions (PPIs) represent important and promising therapeutic targets that are associated with the regulation of various molecular pathways, particularly in cancer. Although they were once considered “undruggable,” the recent advances in screening strategies, structure-based design, and elucidating the nature of hot spots on PPI interfaces, have led to the discovery and development of successful small-molecule inhibitors. In this report, we are describing an integrated high-throughput and computational screening approach to enable the discovery of small-molecule PPI inhibitors of the anti-apoptotic protein, Mcl-1. Applying this strategy, followed by biochemical, biophysical, and biological characterization, nineteen new chemical scaffolds were discovered and validated as Mcl-1 inhibitors. A novel series of Mcl-1 inhibitors was designed and synthesized based on the identified difuryl-triazine core scaffold and structure-activity studies were undertaken to improve the binding affinity to Mcl-1. Compounds with improved *in vitro* binding potency demonstrated on-target activity in cell-based studies. The obtained results demonstrate that structure-based analysis complements the experimental high-throughput screening in identifying novel PPI inhibitor scaffolds and guides follow-up medicinal chemistry efforts. Furthermore, our work provides an example that can be applied to the analysis of available screening data against numerous targets in the PubChem BioAssay Database, leading to the identification of promising lead compounds, fuelling drug discovery pipelines.

## Introduction

Identification and development of novel bioactive small molecules play a key role in basic biological studies, along with drug discovery efforts, by providing chemical tools that lead to new therapeutic targets and potential drugs^1^. The high-throughput screening (HTS) approach has been successfully applied to discover bioactive compounds^2^, particularly for proteins with well-defined binding pockets^3^. However, there is still a need to develop strategies that increase the discovery of small molecules against challenging targets, such as protein-protein interactions (PPIs), transcription factors, and scaffold proteins^4,5^.

PPIs play a crucial role in regulating many biological functions and a variety of diseases, including cancer, are driven by the dysregulation of PPIs^6^. The advances in genomics and proteomics have contributed to a better understanding of PPIs on the functional and structural level, leading to their validation as therapeutic targets. PPIs have been traditionally characterized as “undruggable” targets, due to the major challenge of developing small molecules that potently bind to the relatively flat and large interfaces^7^. Many of these protein interfaces are hydrophobic in nature, so the developed drug leads usually display hydrophobicity themselves, with high molecular weight, low solubility, and poor oral bioavailability, representing unfavourable physicochemical properties for drug candidates^8^. Thus, identification of lead small molecules with chemical diversity and drug-like properties has become a prerequisite for developing effective PPI inhibitors.

The Bcl-2 family of proteins, consisting of anti- (Bcl-2, Bcl-xL, Bcl-w, Mcl-1, Bfl-1, Bcl-B) and pro- (Bax, Bak, Bim, Bad, Bmf, tBid, Noxa, Bik, Puma, Hrk) apoptotic proteins, are major regulators of the intrinsic programmed cell death pathway, and their activity is governed through a network of PPIs between pro- and anti- apoptotic members^9,10^.

**Figure 1:**
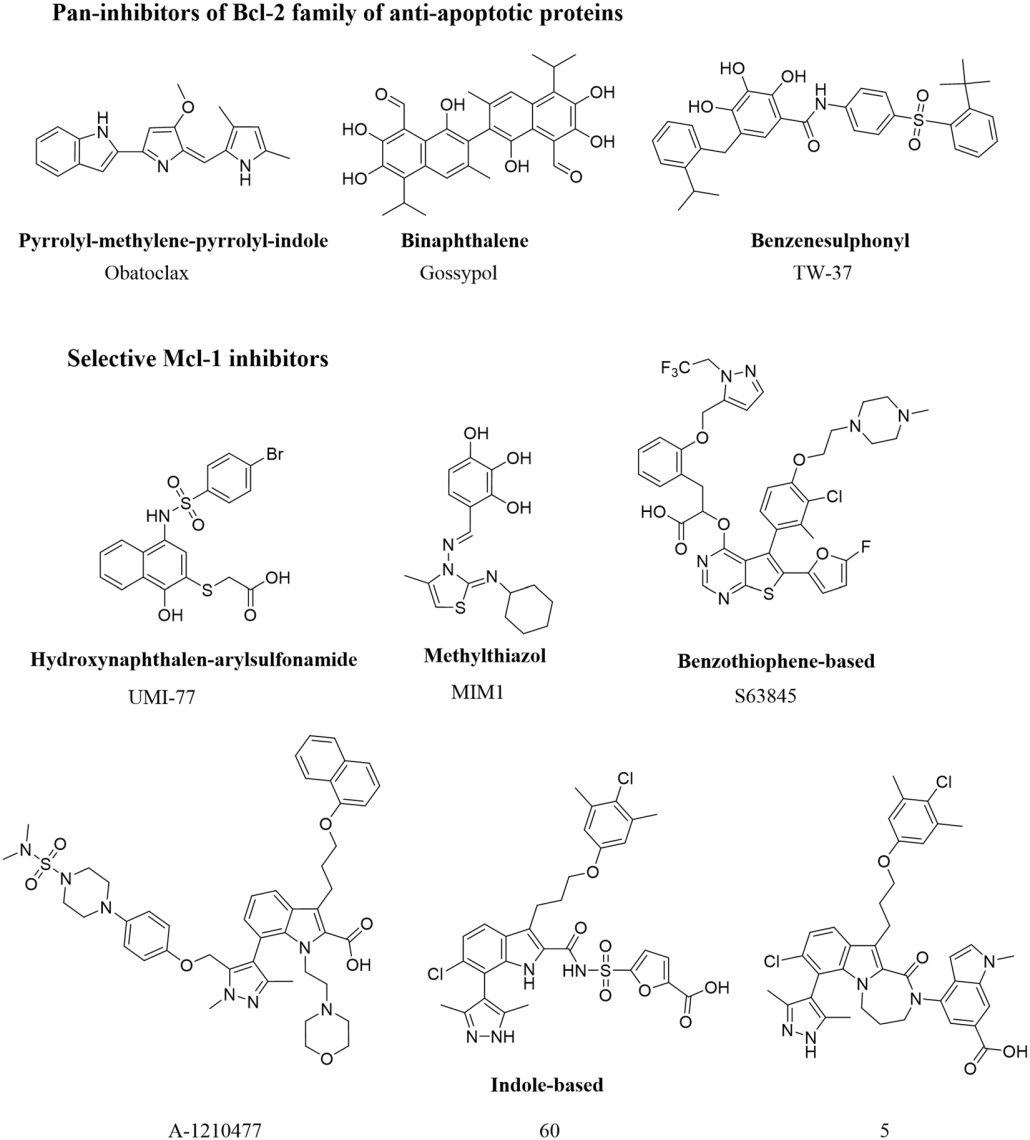
Reported small-molecule Mcl-1 inhibitors with different scaffolds.

Evading apoptosis by cancer cells represents one of the defined cancer hallmarks^11^. Numerous studies have demonstrated the role of anti-apoptotic Bcl-2 proteins in tumour pathogenesis^9^. Inhibiting the function of anti-apoptotic Bcl-2 proteins has become a validated approach for developing novel cancer therapies^10,12,13^, and recently resulted in the FDA approval of the selective Bcl-2 inhibitor, venetoclax (ABT-199)^14^. The anti-apoptotic protein, myeloid cell leukaemia-1 (Mcl-1), is frequently overexpressed in a variety of human cancers, mediating their survival and resistance to chemo- and targeted therapy^15–21^. Mcl-1 has become a well-validated therapeutic target, promoting the development of new anti-cancer therapies^22–24^. Employing different approaches, a number of small molecules with unique scaffolds were identified as pan-inhibitors of the anti-apoptotic Bcl-2 family proteins, along with selective Mcl-1 inhibitors (Fig. 1). Pan-inhibitors include: Obatoclax^25^ and its analogues based on the pyrrolyl-methylene-pyrrolyl-indole scaffold (SC-2001)^26,27^; Gossypol^28^ and its more potent analogues apogossypol (Sabutoclax), BI97C10, and BI112D1^29–31^ with a binaphthalene scaffold, and TW-37 as a second generation benzenesulphonyl derivative of Gossypol^32,33^. Our lab described selective Mcl-1 inhibitors (UMI-77 and compound 21) with hydroxynaphthalen-arylsulphonamide core scaffolds and demonstrated their on-target cellular activity^34^ and efficacy in pancreatic cancer^35,36^ and acute myeloid leukaemia cells^37^. MIM1 is a low micromolar Mcl-1 inhibitor with a methylthaizol scaffold^38^. The AbbVie team and the Fesik group described potent Mcl-1 inhibitors based on the indole-2-carboxylic acid core scaffold^39–43^. Fesik and colleagues reported further modified and optimized series of compounds with extended 2-indole-acylsulphonamides and tricyclic indole diazepinone core^40,41^. A potent Mcl-1 inhibitor, S63845, developed by Servier was recently reported with a thienopyrimidine scaffold and displayed potent *in vitro* and *in vivo* activity^44^.

In this study, we are reporting an integrated high throughput and structure-based virtual screening approach for the discovery of selective Mcl-1 inhibitors with novel chemical scaffolds. Using Mcl-1 as our target protein, we are highlighting the advantages of taking into consideration known structural information to identify hit compounds that mimic conserved interactions, followed by further development and optimization of validated hit compounds. This integrated strategy can be generalized and applied to other targets by analysing deposited results in the PubChem BioAssay Database, which is a public repository for archiving biological tests of small molecules generated through high-throughput screening experiments from more than one million bioactivity assays^45^.

## Results and Discussion

### Integrated screening approach for identifying Mcl-1 inhibitors

We have reported the development and optimization of a dual-readout HTS assay that combines two assay technologies into one system, time-resolved fluorescence resonance energy transfer (TR-FRET) and fluorescence polarization (FP), for the discovery of Mcl-1 protein inhibitors^46^. For the HTS campaign, 102,255 compounds were screened using two different labelled BH3 peptides derived from the Noxa and Bid pro-apoptotic proteins. These two peptides were selected because of their different binding profile and potency against anti-apoptotic proteins. Noxa binds selectively to Mcl-1, while Bid, as an activator, is promiscuous and binds all anti-apoptotic Bcl-2 family proteins, with stronger binding affinity to Mcl-1 in comparison to Noxa^47^. Indeed, Noxa and Bid BH3 labelled peptides yielded a different number of hits that were identified, 875 and 509 compounds, respectively, with 170 common compounds, obtaining the total number of 1,214 hit compounds. These results were deposited into the PubChem BioAssay Database (AID 1417, Dose Response Confirmation for Mcl-1/Noxa Interaction Inhibitors, and AID 1418, Dose Response Confirmation for Mcl-1/Bid Interaction Inhibitors).

Structural studies of the Bcl-2 family of proteins and their complexes highlighted and defined the interactions between anti- and pro-apoptotic proteins. These studies showed that the Bcl-2 homology 3 (BH3) domain of the pro-apoptotic proteins binds as an amphipathic α-helix in a hydrophobic groove formed from residues in the BH1, BH2 and BH3 motifs of the anti-apoptotic proteins, known as the BH3-binding groove^10,48,49^. Although pro-apoptotic proteins share sequence homology in the BH3 region and bind to the same hydrophobic BH3 pocket, each BH3-only protein has different binding potency and selectivity for the anti-apoptotic proteins^50^. To take advantage of these known structural characterizations of the interactions between Mcl-1 and pro-apoptotic proteins, we integrated a computational structure-based searching strategy and applied it in the selection of the most promising hit compounds for the follow up biochemical and biophysical secondary assays. This strategy also allowed for the exclusion of false positives, such as aggregators, protein reactive, or compounds that interfere with the assay readout^51,52^.

**Figure 2:**
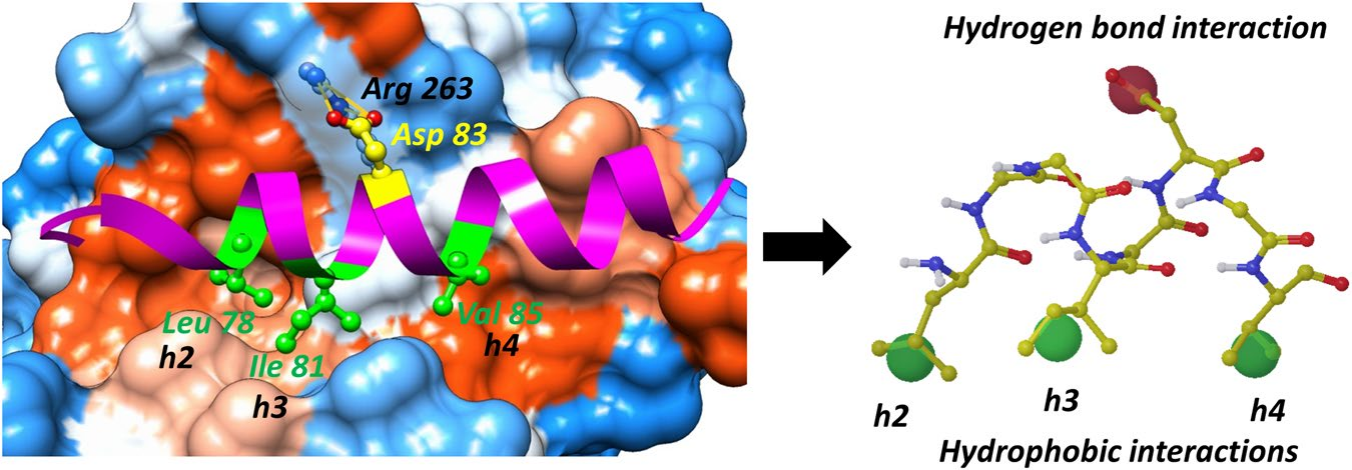
Structure-based pharmacophore model extracted from the complex of Noxa BH3 peptide with Mcl-1 (PDB entry 2NLA).

**Figure 3:**
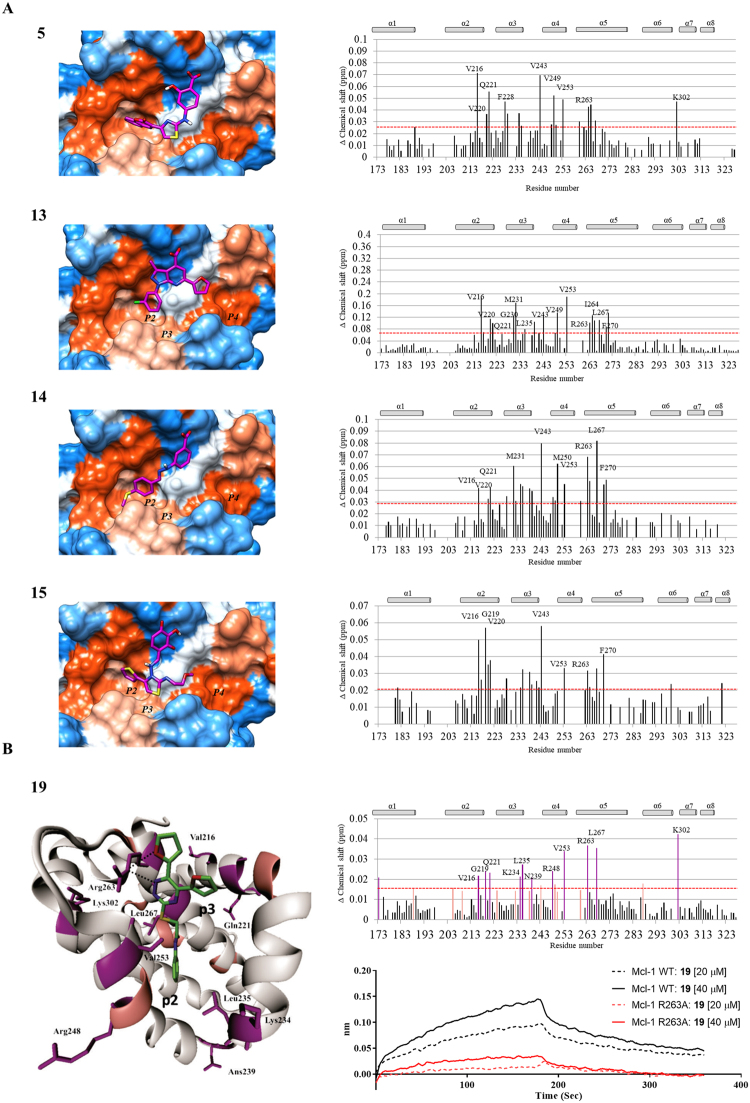
Putative binding modes and biophysical characterization of identified hits. (**A**) Left: Computational predicted binding poses of the compounds in Mcl-1 binding site using the mNoxa BH3 peptide-bound Mcl-1 crystal structure (PDB: 2NLA). The Mcl-1 protein is colour coded to illustrate hydrophobic (orange) and hydrophilic (blue) surfaces. Right: Plot of chemical shift changes of Mcl-1 amide in the presence of the corresponding compounds (2 equivalents) as a function of Mcl-1 residue numbers, obtained by the HSQC NMR analysis. The red dashed line represents the significance threshold (1 SD above the average chemical shift perturbations). (**B**) The predicted binding mode of **19** to Mcl-1 with residues colored according to the chemical shift intensity. Significant shifts (>0.014 ppm) are represented with purple, while moderate shifts are represented with pink. (**B**) Binding curves of compound **19** obtained in a BLI-based assay using immobilized wild type (WT) and Mutant Mcl-1 (R263A) (black and red colored, respectively).

For this purpose, a high-resolution co-crystal structure of the Noxa BH3 peptide in complex with Mcl-1 (PDB ID: 2NLA) was used, which provided insights into the essential pharmacophore (Fig. 2)^53^. Similar to other pro-apoptotic proteins, three hydrophobic interactions involving the conserved residues, Leu 78, Ile 81 and Val 85 (h2, h3, and h4 respectively), and a hydrogen bond formed between conserved charged Asp 83 in the BH3 Noxa peptide and Arg 263 in the Mcl-1 protein, are crucial for interactions between Noxa and Mcl-1. In order to identify compounds that bind into the Mcl-1 BH3 binding groove and mimic these conserved interactions, the identified 1,214 hits were analysed by *in silico* and molecular docking studies. Based on the Noxa pharmacophore model, the grid box of Mcl-1 for the docking studies was defined by the Phe 270 (in the P2 pocket), Val 220 (in the P3/P4 pocket), Val 216 (in the P4 pocket), as well as the charged Arg 263 residue. The 1,214 compounds obtained from the HTS assays (AID 1417 and 1418) were first analysed using Glide 5.6 Standard Precision (SP) as a docking tool, followed by the Induced Fit Docking (IFD) protocol, in order to confirm the binding poses and introduce the flexibility and adaptability of the anti-apoptotic Bcl-2 family proteins^54^. In the post-process analysis, the obtained docking poses were visually inspected in order to select the most promising compounds for further testing. Besides having a high docking score and a reasonable binding pose, additional compound selection criteria was the ability to mimic at least two conserved interactions in the BH3 binding site based on the pharmacophore model (Fig. 2, Supplementary Table S1 and Fig. S1). Through the evaluation of binding modes and Mcl-1 protein interactions, 67 compounds were selected. All selected compounds formed the conserved hydrogen bond with Arg 263, serving as an anchor to bind the BH3 binding site of the Mcl-1 protein. Interestingly, the majority of the selected compounds mimicked the h2 and h3 hydrophobic interactions and occupied the corresponding P2 and P3 pockets (Fig. 3, Supplementary Figs S1 and S2). This was an important finding, knowing that the interactions through the conserved h3 hydrophobic residue are critical for Mcl-1 selectivity^55^. 36 commercially available compounds that covered diverse chemical space were selected for further characterization as Mcl-1 inhibitors.

### Experimental validation through biochemical and biophysical assays

To confirm and validate the most promising Mcl-1 inhibitors, 36 identified and purchased compounds were characterized with biochemical and biophysical methods, including FP- and surface plasmon resonance (SPR)-based competitive binding assays, using fluorescent labelled Bid and biotin labelled Bim BH3 peptides, respectively, as the competing ligands^35,37^. The direct binding to Mcl-1 and mapping of compounds’ binding site was determined by Bio-Layer Interferometry (BLI) and ^1^H,^15^N heteronuclear single and quantum correlation (HSQC) NMR spectroscopy^37^. Nineteen compounds were confirmed and showed binding to Mcl-1 protein by using these different binding platforms and assays (Table 1). The remaining seventeen compounds were considered unconfirmed for several reasons, some didn’t show binding in the tested concentration range (up to 100 μM), for others, the obtained binding data showed significantly lower potency in comparison with the dose-response experiments from the HTS campaign or the binding data was not consistent in all biochemical and biophysical binding assays (Supplementary Table S2).

**Table 1:** Chemical structures and binding IC_50_ values obtained by competitive FP- and SPR-based assay of identified hit Mcl-1 inhibitors confirmed by HSQC NMR studies.

Cpd	Structure	FP IC_50_ [µM]	SPR IC_50_ [µM]	AID 1417 IC_50_ [µM]^a^	AID 1418 IC_50_ [µM]^b^	Binding to Bfl-1 FP IC_50_ [µM]
**1**		2.6 ± 0.4	3.8 ± 1.8	1.5	0.9	5.8 ± 1.8
**2**		5.9 ± 1.2	8.8 ± 2.7	7.2	N.D.^c^	26.7 ± 4.8
**3**		9.6 ± 2.5	21.3 ± 5.4	14.3	N.D.	2.5 ± 0.2
**4**		2.4 ± 1.1	4.5 ± 0.3	2.4	N.D.	N.D.
**5**		39.4 ± 4.3	32.0 ± 7.7	5.6	N.D.	>100
**6**		23.7 ± 3.2	18.7 ± 11.5	4.8	N.D.	N.D.
**7**		5.2 ± 1.9	11.7 ± 4.1	N.D.	26.8	>100
**8**		16.5 ± 1.4	4.9 ± 2.4	7.6	N.D.	N.D.
**9**		1.2 ± 0.2	2.7 ± 0.4	2.0	0.7	N.D.
**10**		5.5 ± 1.7	3.7 ± 0.2	2.2	N.D.	22.9 ± 2.9
**11**		9.4 ± 3.8	2.8 ± 0.9	3.7	N.D.	>100
**12**		N.A.^d^	10.7 ± 4.2	1.7	N.D.	N.A.^d^
**13**		17.9 ± 2.1	66.1 ± 8.0	9.6	N.D.	96.0 ± 23.2
**14**		1.9 ± 0.6	1.7 ± 0.5	2.2	N.D.	N.D.
**15**		2.7 ± 1.0	6.5 ± 2.2	5.2	N.D.	>100
**16**		39.0 ± 9.4	20.0 ± 1.7	4.3	N.D.	>100
**17**		23.7 ± 1.6	14.9 ± 0.7	N.D.	0.5	N.D.
**18**		11.1 ± 1.8	50.0 ± 9.3	7.2	N.D.	N.D.
**19**		13.8 ± 1.4	13.4 ± 0.2	3.1	N.D.	35.6 ± 8.6

^a^The IC_50_ value deposited into PubChem BioAssay (AID 1417: Dose Response Confirmation for Mcl-1/Noxa Interaction Inhibitors).

^b^The IC_50_ value deposited into PubChem BioAssay (AID 1418: Dose Response Confirmation for Mcl-1/Bid Interaction Inhibitors).

^c^N.D. Not determined.

^d^N.A. The compound interferes with the FP assay.

FP and SPR-based binding data demonstrated that all nineteen compounds were able to compete with Bid and Bim BH3 peptides with micromolar IC_50_ values, ranging from 1 to 50 μM, similar to the binding affinities obtained during the dose-response screening assays (AID 1417 and 1418) (Table 1). Consistent with the competitive binding results, all confirmed compounds showed strong chemical shift perturbations, demonstrating the direct binding to the Mcl-1 protein. Analysing the ligand-induced chemical shifts and identifying the residues affected by ligand binding, the binding site of tested inhibitors was mapped. Using this information in combination with docking studies, the predicted binding models of the inhibitors in complex with Mcl-1 were obtained. In Fig. 3, the binding modes of selected validated hit compounds **5**, **13**, **14**, **15**, and **19** are presented, along with the corresponding chemical shift perturbations obtained from the ^1^H,^15^N HSQC NMR studies. For the rest of the active compounds, the same information is provided in the Supplementary Fig. S2. The predicted binding modes of these compounds in complex with Mcl-1 revealed the occupation of two hydrophobic pockets, mimicking conserved hydrophobic residues Leu 78 (h2) and Ile 81 (h3) in the BH3 region of the Noxa peptide (Fig. 3). Mcl-1 residues involved in these hydrophobic interactions with the chemical moieties of the compounds include Val 243, Met 250, Val 253, and Phe 270 from the P2 pocket, and Met 231 and Val 220 from the P3 pocket. The conserved hydrogen bond interaction with the charged Mcl-1 Arg 263 residue was observed in all compounds, mimicking the conserved Asp in the pro-apoptotic proteins (Fig. 3 and Supplementary Fig. S2). To demonstrate the importance of the hydrogen bond interactions between the inhibitors and the Arg 263 on Mcl-1, we prepared an R263A mutant of Mcl-1. As was expected, the positive controls, Flu-Bid and Noxa BH3 peptides didn’t show binding in the FP- and BLI-based binding assays, respectively (Supplementary Fig. S3A and B). Using the BLI platform, we tested and compared the binding of identified compounds against wild-type and mutant R263A Mcl-1 proteins (Fig. 3B and Supplementary Fig. S3C). Mcl-1 inhibitors, **5**, **13**, **14** and **19**, demonstrated no appreciable binding to the mutant Mcl-1 protein, indicating their interactions with Arg 263, most likely through a hydrogen bond. These results, together with the chemical shift perturbations detected in the HSQC NMR experiments, align well with the predicted *in silico* binding poses and provided conclusive evidence that the identified compounds bind to the BH3-binding groove of Mcl-1 protein. As a validation of the applied integrated HTS and computational screening approach, it is important to be pointed out that this strategy successfully identified compounds that were already reported as Mcl-1 inhibitors. For example, the scaffold of compound **8** closely resembles the class of selective Mcl-1 inhibitors developed by our group^35,37^, and compound **15** possess a similar scaffold to the reported Mcl-1 inhibitor, MIM1^38^.

The BH3-only pro-apoptotic protein, Noxa, binds selectively to the Mcl-1 and Bfl-1 (also known as Bcl2A1 or A1) anti-apoptotic proteins, therefore we tested the identified and confirmed hit compounds for their binding to Bfl-1 (Table 1). Interestingly, compounds showed a wide range of binding affinities to Bfl-1, from low micromolar to no binding up to 100 µM. Some compounds, like **1** and **3**, showed similar binding potency against both Mcl-1 and Bfl-1. Most of the tested compounds showed lower binding to Bfl-1 in comparison to Mcl-1 (**2**, **5**, **10**, **13** and **19**), while compounds **7**, **11** and **15**, didn’t show binding in the tested concentration. These results demonstrate that the Bfl-1 BH3 binding pocket is different from Mcl-1, providing evidence that it should be possible to obtain selective Bfl-1 inhibitors versus other Bcl-2 family members.

In order to facilitate the selection of a hit compound for further development, in addition to biochemical and biophysical characterization, we also used a cell-based assay for screening of our identified and confirmed hit compounds. It is well established that Bax and Bak play crucial roles as effectors in the intrinsic apoptotic pathway and are required for release of cytochrome c from the mitochondria and cell death execution^56^. On-target anti-apoptotic Bcl-2 protein family inhibitors are expected to induce cell death in a Bax/Bak-dependent manner^57^. Therefore, we used wild-type and Bax/Bak double knockout (DKO) mouse embryonic fibroblasts (MEFs) to test identified hits (Supplementary Fig. S4). These results showed that the majority of compounds didn’t display significant effect in either cell line. Importantly, compounds **6**, **15** and **19** exhibited selectivity in inducing cell death only in wild-type MEFs, without significant cell death induction in the DKO cells. The obtained results for compound **15**, an analogue of a reported Mcl-1 inhibitor, is consistent with reported findings^57^. Interestingly, compounds **7** and **9** lacked specificity and induced cell death in both wild-type and DKO cell lines.

### Hit-to-lead optimization of the validated hit compound 19

The integrated screening approach identified a variety of chemical scaffolds as Mcl-1 inhibitors. Based on the overall binding data against anti-apoptotic proteins, together with the ability to induce Bax/Bak-dependent cell death, compound **19** with a difuryl-triazine scaffold, was selected for further optimization and evaluation via structure-activity relationship (SAR). In addition, the triazine scaffold is one of the well-known and important heterocycles, with a broad range of biological activities, thus representing a valuable core structure for the development of bioactive compounds^58^.

**19** binds the BH3 binding groove of the Mcl-1 protein and competes with Bid and Bim BH3 peptides, with the IC_50_ value of 13 µM (Tables 1 and 2). The HSQC NMR spectroscopy confirmed the direct binding, showing concentration-dependent perturbations of the backbone amide residues. The observed NMR shifts of the residues in the P2 pocket, Lys 234, Leu 235, and Val 253, were in line with the generated docking model of **19**, where the terminal phenyl ring occupies this pocket (Fig. 3B). The docking model of **19** revealed three hydrogen bonds formed by the oxygen atom of one furan ring, one nitrogen atom of the triazine ring, and residues, Arg 263 and Asn 260 of Mcl-1, mimicking the conserved Asp in pro-apoptotic proteins. Indeed, the HSQC NMR spectrum of the **19**:Mcl-1 complex showed that Arg 263 has a significant chemical shift. The sulphur-acetamide chain does not have direct interactions with the protein of Mcl-1, playing the role as a linker (Fig. 3B). To gain further insights into the SAR of compound **19** and probe the proposed binding mode, we proceeded with medicinal chemistry optimization of a novel series of 1,2,4-triazine-based Mcl-1 inhibitors. Our goal was to primarily examine the importance of the furan interaction with Arg 263, and to design and synthesize analogues to optimize hydrophobic interactions with the Mcl-1 P2 binding pocket in order to improve binding affinity.

**Table 2:** SAR studies of furan moieties at R1 position and R2 substituents.


Cpd	R1	R2	FP IC_50_ [µM]	FP *K*_i_ [µM]	SPR IC_50_ [µM]	TR-FRET IC_50_ [µM]
**19**			13.8 ± 1.4	3.2 ± 0.3	13.4 ± 0.2	11.9 ± 3.7
**25**			>100	>25	>100	>100
**26**			>20	>5	>100	N.D.
**27**			>50	>12.5	>100	N.D.
**28**			5.0 ± 0.3	1.2 ± 0.1	14.3 ± 2.0	5.5 ± 1.8
**29**			2.3 ± 0.7	0.5 ± 0.2	11.6 ± 1.1	N.D.
**30**			33.1 ± 7.7	7.8 ± 1.8	47.4 ± 3.9	N.D.
**31**			6.9 ± 1.9	1.6 ± 0.4	N.D.	9.0 ± 3.8
**32**			>100	>25	>100	>80
**33**			>100	>25	>100	N.D.
**34**			8.1 ± 0.8	1.9 ± 0.2	12.5 ± 1.7	11.6 ± 4.0
**35**			2.5 ± 0.1	0.6 ± 0.1	5.1 ± 2.3	5.0 ± 2.7
**36**			2.5 ± 0.6	0.6 ± 0.1	4.2 ± 0.4	5.1 ± 1.5
**37**			5.3 ± 0.5	1.2 ± 0.1	N.D.	N.D.
**38**			2.6 ± 0.3	0.6 ± 0.1	N.D.	N.D.
**39**			5.1 ± 0.5	1.2 ± 0.1	6.3 ± 0.6	7.1 ± 2.8
**40**			4.1 ± 1.5	1.0 ± 0.4	7.3 ± 2.1	7.9 ± 4.7
**41**			4.4 ± 0.5	1.0 ± 0.1	1.9 ± 0.7	12.7 ± 4.5

**Figure 4:**
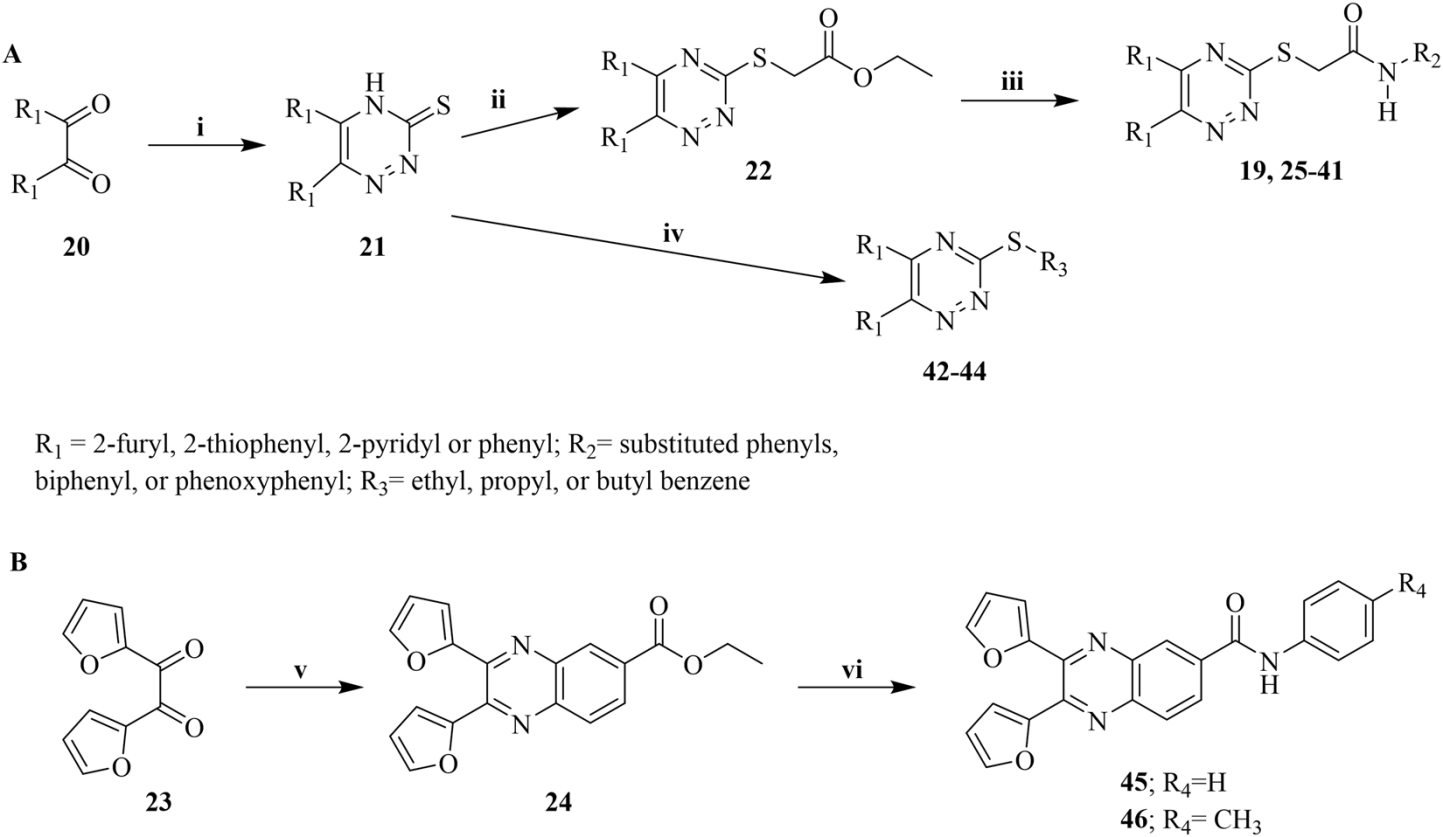
Synthetic Route for (**A**) 5, 6-disubstitued-1,2,4-triazine and (**B**) 2,3-difuryl-quinoxaline analogues. Reagents and conditions: (i) hydrazinecarbothioamide, EtOH:DMF (2:1), K_2_CO_3_, AcOH, 8 h, reflux; (ii) ethyl 2-chloroacetate, dry acetone, K_2_CO_3_ reflux, 1.5 h; (iii) R_2_-NH_2_, Me_3_Al, Benzene, reflux, 2h-48h, 46–93%; (iv) Aralkyl chloride, dry acetone, K_2_CO_3_ reflux, 1.5 h, 21–47%; (v) EtOH/DMF, K_2_CO_3_, methyl 3,4-diaminobenzoate; (vi) Me_3_Al, Benzene, benzylamine or p-methyl benzylamine, 54–76%.

The synthesis of **19** and its analogues was performed following the general synthetic route presented in Fig. 4. The synthesis of **19** and **25–44** was accomplished by condensation of commercially available diketo compounds **20** and thiosemicarbazide, which provided 1,3,5-triazine thiols **21** (Fig. 4A)^59,60^. Subsequent nucleophilic addition of the aromatic thiols **21** with ethyl chloroacetate or aralkyl chloride afforded the corresponding esters **22** and desired compounds **42–44**, respectively. The resulting esters readily underwent ester-amide exchange reaction upon treatment with substituted anilines in the presence of trimethylaluminum to afford the desired compounds **19** and **25–41**^61,62^. Similarly, synthesis of 2,3-difuryl-quinoxaline derivatives **45** and **46** was carried out by condensation of 1,2-di(furan-2-yl)ethane-1,2-dione **23** with methyl 3,4-diaminobenzoate to provide 2,3-difuryl-quinoxaline ester **24**, which in turn was coupled with amines to provide corresponding amides **45** and **46** (Fig. 4B). Characterization data of the synthesized compounds are available in the supplementary material.

Initial optimization efforts focused on systematic modifications to the furan to explore the conserved interaction with Arg 263 on the Mcl-1 protein (Table 2). Replacement of furan rings (denoted as R_1_) with thiophene **25**, phenyl **26**, and pyridine rings **27**, resulted in a significant loss in binding affinity (>100 μM), confirming the importance of the hydrogen bond. This is consistent with our docking and binding studies using R263A mutant Mcl-1, which demonstrated the crucial role of Arg 263 in binding of this scaffold (Fig. 3B). While in **26**, the phenyl rings don’t have hydrogen bond acceptors and it was expected to see loss of the binding, the reason why we are seeing the same phenomena with compounds **25** and **27** is probably due to lack of the preferred geometry for hydrogen bond interactions between the Mcl-1 Arg 263 and the hydrogen bond acceptors in thiophene and pyridine rings, respectively^63,64^.

Analysing the available Mcl-1 structural data, it was established that its P2 binding pocket is the deepest and largest in volume compared to the other pockets of the BH3 binding groove. In the next step, the optimization efforts and modifications were focused on the distal phenyl group, which binds into P2 pocket based on the docking and HSQC NMR studies. A series of compounds were designed and synthesized to explore variations in substituent size, lipophilicity, and polarity of introduced substituents on the phenyl group and their impact on the binding affinity against Mcl-1 (Table 2). The para substitution on the benzene ring, showed clear preference for hydrophobic substituents, including short alkyl groups, like ethyl and propyl in compounds **28** (IC_50_ = 5.0 ± 0.3 µM) and **29** (IC_50_ = 2.3 ± 0.7 µM), as well as larger substituents, phenyl in **35** and benzyl in compound **36**, showing similar potency with IC_50_ values of 2.5 μM. Interestingly, incorporating of electron donating groups, such as methoxy (**30**, R_2_ = 4-OMe-Ph), primary amine (**33**, R_2_ = 4-NH_2_-Ph), and tertiary amine (**32**, R_2_ = 4-N(Me)_2_-Ph), resulted in significant loss of binding affinity with IC_50_ values of 33 μM and >100 μM, respectively. These results confirmed that the P2 pocket can accommodate larger size substituents, primarily hydrophobic in their nature, consistent with reported Mcl-1 inhibitors^37,39,42,44^. Changing the position of the substituents from para to meta position on the distal phenyl ring, such as ethyl and isopropyl groups in **37** and **38** respectively, gave binding affinities in line with the corresponding compounds, **28** and **29**. Similar potency was also observed by introducing a CF_3_ group in the meta position (**39**, IC_50_ = 5.1 ± 0.5 µM). In an effort to explore the tolerance for larger hydrophobic groups on the meta and ortho positions, we synthesized **40** and **41** respectively, which gave 2- to 3-fold decrease in the potency. Overall, these results showed a clear preference for hydrophobic substituents at the 4-position of the distal phenyl ring.

Given the data obtained from the SAR of the distal phenyl group, we sought to further explore SAR of the amide chain linker and the triazine core scaffold (Table 3).

**Table 3:** SAR studies of R3 substituents and core scaffold.


Cpd	R3	R4	FP IC_50_ [µM]	FP *K*_i_ [µM]	SPR IC_50_ [µM]	TR-FRET IC_50_ [µM]
**42**		—	1.5 ± 0.3	0.4 ± 0.1	N.D.	2.4 ± 0.1
**43**		—	2.2 ± 0.2	0.5 ± 0.1	N.D.	5.2 ± 0.1
**44**		—	1.4 ± 0.2	0.3 ± 0.1	N.D.	2.5 ± 0.1
**45**	—		>50	>12.5	N.D.	N.D.
**46**	—		67.1 ± 9.1	15.8 ± 2.3	N.D.	N.D.

Generally, substituting the amide with a carbon linker, as exemplified by analogues **42** to **44**, resulted in significant 7- to 9-fold improvement in comparison with the hit compound **19**. These binding results demonstrated that the flexibility of the linker is very important for enhanced binding to Mcl-1 protein, probably by better accommodation of the distal phenyl ring into the P2 hydrophobic pocket. Indeed, these findings are consistent with the reported indole-based Mcl-1 inhibitors developed by fragment merging^39^. This study demonstrated that linking the fragments through a four-atom linker yielded the most potent selective Mcl-1 inhibitors. Furthermore, X-ray complexed structures showed that the flexible carbon linker provided favourable hydrophobic contacts in the P2 pocket^39^.

The importance of the triazine core and tolerance for its modification was evaluated via the synthesis of analogues, **45** and **46**, where the core was replaced with a quinoxaline ring. In both cases, inhibition was abrogated with an IC_50_ > 50 µM. Overall, the obtained SAR results emphasize the 1,2,4-triazine and its derivatives as a promising scaffold and series of Mcl-1 inhibitors for further development. The binding affinity results and obtained SAR were confirmed with different competitive assays: biochemical, including FP and TR-FRET based assays, as well as the biophysical SPR platform.

The identified and validated hit compound **19** showed selectivity for Mcl-1 (K_i_ = 3.2 µM) and Bfl-1 (K_i_ = 4.9 µM) in comparison with Bcl-2 (K_i_ > 25 µM) and Bcl-xL (K_i_ > 20 µM) (Supplementary Table S3). In order to test if the chemical modifications interfere with the selectivity profile of **19**, several novel representative compounds with improved binding affinity to Mcl-1 were assessed for their binding profile. Encouragingly, the results confirmed that all tested compounds, similarly as the initial hit compound, preserve the selectivity profile, showing similar binding affinity to Mcl-1 and Bfl-1 and no binding to Bcl-2 and Bcl-xL, up to 100 µM. Interestingly, compound **39** is the most selective Mcl-1 inhibitor (K_i_ = 1.2 µM) showing 8-, >20-, and >17-fold lower binding affinity to Bfl-1 (K_i_ = 10.4 µM), Bcl-2 (K_i_ > 25 µM) and Bcl-xL (K_i_ > 20 µM), respectively.

### Biological characterization of novel Mcl-1 inhibitors

**Figure 5:**
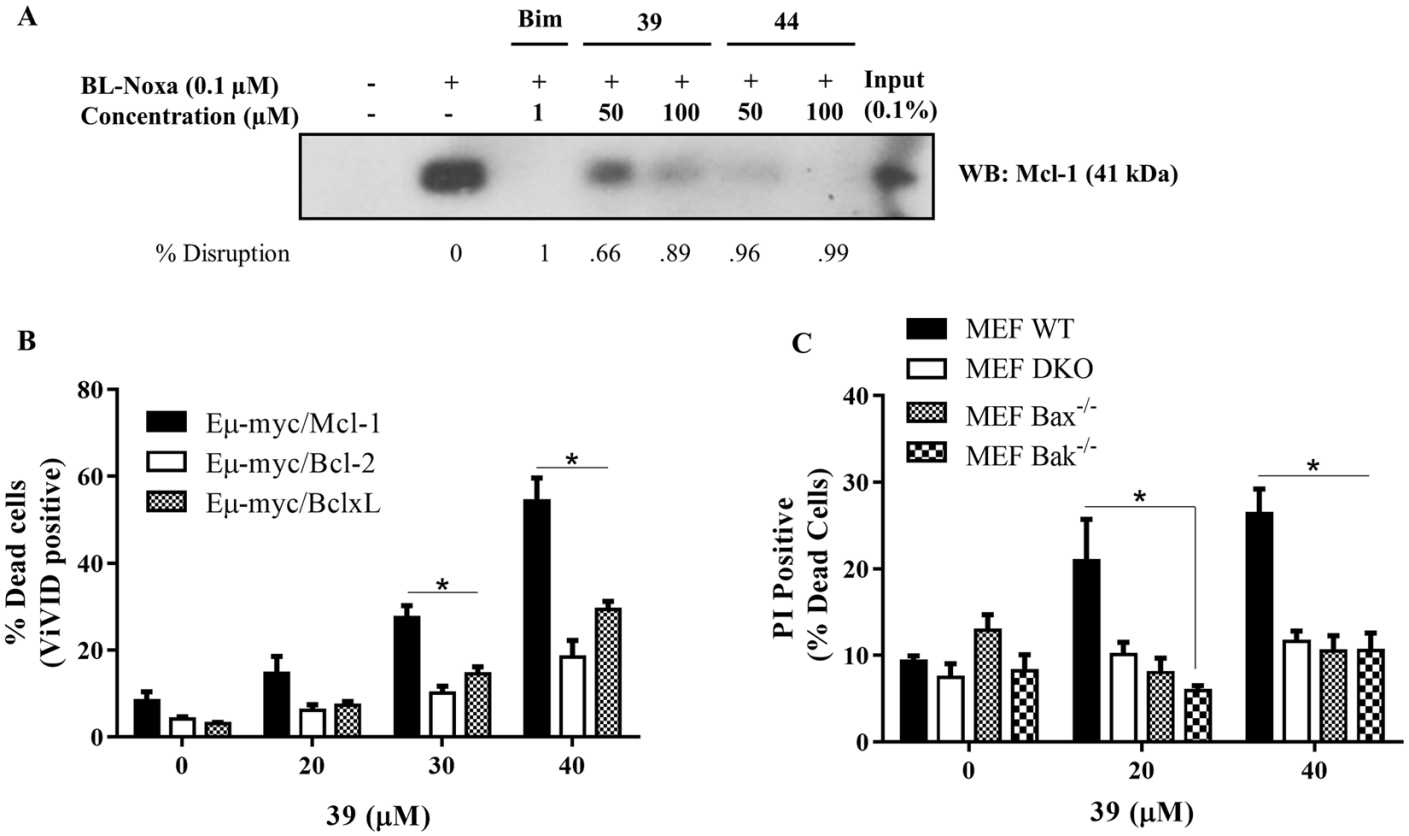
Optimized Mcl-1 inhibitors demonstrate on-target cellular activity. (**A**) **39** and **44** interact with endogenous Mcl-1 protein and disrupt the interaction with BL-Noxa; (**B**) **39** shows selective killing of Eµ-myc lymphoma cells overexpressing Mcl-1 versus Eµ-myc Bcl-2 or Eµ-myc Bcl-xL lymphomas (**C**) **39** induces Bax/Bak-dependent cell death of MEFs. (*) is *p* < 0.05.

To confirm the binding of optimized inhibitors to endogenous Mcl-1, we employed a pull-down assay, using biotin-labelled Noxa (BL-Noxa) and whole cell lysate from the human breast cancer cell line, 2LMP. As shown in Fig. 5A, Mcl-1 was pulled down by BL-Noxa and, as expected, the positive control Bim BH3 peptide disrupted the interaction between BL-Noxa and Mcl-1. Incubation with **39** and **44** resulted in the dose-dependent blocking of the binding of BL-Noxa to Mcl-1, with **44** showing more potent disruption compared to **39** at the same doses tested, consistent with their binding affinities (Tables 2 and 3).

In order to explore the target specificity in the context of cellular activity of this novel series of Mcl-1 inhibitors, engineered Eµ-myc lymphoma model cell lines were used which overexpress and depend on individual anti-apoptotic Bcl-2 proteins for survival^65^. Lymphoma cells overexpressing Mcl-1, Bcl-2 or Bcl-xL were treated with increasing concentrations of inhibitors for 15 h and cell viability was determined by flow cytometry. As was expected, Eµ-myc cells overexpressing Mcl-1 were significantly more sensitive to **39** in a concentration-dependent manner, in comparison with Eµ-myc cells overexpressing Bcl-2 or Bcl-xL (Fig. 5B). This confirms that **39** selectively kills Mcl-1 dependent cell lines, with minimal effect on cell lines that depend on Bcl-2 and Bcl-xL, consistent with its selective binding profile (Supplementary Table S3). Providing further validation of these model cell lines, the reported selective Mcl-1 inhibitor, S63845, showed the same profile by selectively killing the Eµ-myc/Mcl-1 cell line (Supplementary Fig. S5B). Several representative compounds were tested and they all showed the killing of only Mcl-1 dependent Eµ-myc cells, but not Bcl-2 dependent cells (Supplementary Fig. S5A). However, although compound **44** showed stronger *in vitro* binding and disruption of the interaction between BL-Noxa and endogenous Mcl-1 protein in the pulldown assay, **39** exhibited better potency in the cell-based assay.

MEF cells, including the wild-type, DKO, and both Bax^−/−^ and Bak^−/−^ single knockouts (Supplementary Fig. S6A) were treated with compound **39** to investigate the role of Bax/Bak proteins in cell death induction, as was previously done to validate compound **19** and other identified hits (Supplementary Fig. S4). As expected, treating wild-type MEFs with **39** for 15 h resulted in a dose-dependent increase in cell death, as shown in Fig. 5C. The knockout of either or both Bax and Bak rescued the cells from the effect of **39**, indicating that the presence of both Bax and Bak is necessary to induce cell death by the difuryl-triazine-based Mcl-1 inhibitor, **39**. Similar results were obtained with S63845 (Supplementary Fig. S6B). Taken together, our data support a mechanism in which **39** selectively inhibits endogenous Mcl-1 and induces on-target Bax/Bak-dependent cell death.

In summary, we described a successful integrated screening approach to identify compounds with unique and diverse chemical scaffolds suitable for further development and optimization as selective Mcl-1 inhibitors. For this purpose, we performed a biochemical target-based HTS campaign using Noxa and Bid as labelled probes, followed by structure-based screening using ligand docking and pharmacophore model based on the experimentally determined complex between Mcl-1 and the pro-apoptotic protein, Noxa (PDB:2NLA). Nineteen new chemical scaffolds were confirmed as Mcl-1 binders, with the IC_50_ values in the low micromolar range, yielding novel chemical space suitable for subsequent optimization. Using hit compound **19** with the difuryl-triazine core, a series of inhibitors was designed and synthesized, leading to selective Mcl-1 inhibition with improved binding potency and on-target cellular activity. These results confirmed that the integrated screening approach, followed by medicinal chemistry efforts, revealed a promising class of compounds for further development as selective Mcl-1 inhibitors. Furthermore, this approach also outlines how *in silico* screening complements HTS and results in identifying novel small-molecule PPI inhibitors. This study provides valuable guidance for analysing the PubChem BioAssay Database, by incorporating known structural features of the investigated target and identifying privileged scaffolds for drug discovery and development. It is important to be pointed out that many novel computational methodologies, tools, and databases have been developed and implemented in drug discovery processes, such as homology modelling, ligand fingerprint methods, predicting drug-target interaction, toxicity and carcinogenicity, and synergistic drug combinations^66–71^. Recently, the OncoPPi portal was established as a resource to analyse cancer-related PPIs and facilitate identification of therapeutic targets for drug discovery^72,73^. All these available computational resources and methods are increasing the efficiency and success of the identification and development of new biologically active compounds and are becoming essential components of drug discovery programs.

## Methods

### Molecular Modelling

The chemical structures of the 1,384 compounds from the two performed HTS campaigns AIDs 1417 and 1418 were downloaded from PubChem Bioassay database as two sdf files. After removing 170 duplicated compounds, LigPrep 2.5 of the Schrödinger suite was employed to convert them into 3D structures. Glide 5.6 SP of the Schrödinger suite was used to dock the remaining 1,214 compounds into the Mcl-1 active site^74,75^. The PDB ID of the employed Mcl-1 structure is 2NLA which also contains bound BH3-only peptide Noxa. At the same time, Schrödinger’s IFD was also employed for the docking studies to consider the flexibility of the Mcl-1 protein. Schrödinger’s IFD predicts ligand binding modes and concomitant structural changes in the protein by combining Glide (the docking program of Schrödinger) and the refinement module in Prime (the protein structure prediction program of Schrödinger). Its main application is to generate an accurate complex structure for a ligand known to be active but that cannot be docked in a rigid structure of the receptor. IFD was used in our study because it incorporates the protein flexibility as well as the ligand flexibility, which is important for accurate docking, especially when the protein is showing an increased level of flexibility also experimentally. The docking protocol that was used for the IFD studies consists of the following steps: (1) Constrained minimization of the protein with an RMSD cutoff of 0.18 Å. (2) Initial Glide docking of the ligand using a softened potential (Van der Waals radii scaling). (3) One round of Prime side-chain prediction for each protein/ligand complex, on residues within a defined distance of any ligand pose. (4) Prime minimization of the same set of residues and the ligand for each protein/ligand complex pose. (5) Glide re-docking of each protein/ligand complex structure within a specified energy of the lowest energy structure. (6) Estimation of the binding energy (IFDScore) for each output pose. In our study, all docking calculations were run in the extra precision (XP) mode of Glide. During both of the docking studies, the centre of the grid box of the Mcl-1 was defined by the Val 249 (in h1), Phe 270 (in h2), Val 220 (in h3/h4) and Val 216 (in h4). The size of the grid box was set to 15 Å. Default values were used for all other parameters.

### Fluorescence polarization-based binding assay

FP-based binding assays were developed and optimized to determine the binding affinities of inhibitors to the recombinant Mcl-1, Bcl-2, Bcl-xL, and Bfl-1, proteins. The obtained *K*_d_ values of fluorescent labelled Bid were 3.0 ± 0.1 nM to Mcl-1, 18.4 ± 1.4 nM to Bcl-2, 20.0 ± 2.3 nM to Bcl-xL, and 0.3 ± 0.01 nM to Bfl-1. For testing of purchased compounds and based on the Kd values, the concentrations of the proteins used in the competitive binding experiments were 20 nM of Mcl-1 in assay buffer (50 mM Tris pH 7.4, 50 mM NaCl and 0.005% Tween 20). For testing the synthesized library of compounds based on compound **19**, we used 10 nM of Mcl-1 with Flu-Bid, 60 nM of Bcl-2, 80 nM of Bcl-xL and 2 nM Bfl-1 with FAM-Bid in assay buffer (20 mM potassium phosphate, pH 7.5; 50 mM NaCl, 1 mM EDTA and 0.05% Pluronic F68). The fluorescent Bid probe was fixed at 2 nM for all assays, except for Bfl-1, where 1 nM was used. 5 μL of the tested compound in DMSO and 120 μL of protein/probe complex in the assay buffer were added to assay plates (Corning #3792), incubated at room temperature for 3 h, and the polarization values (mP) were measured at an excitation wavelength of 485 nm and an emission wavelength of 530 nm using the plate reader Synergy H1 Hybrid, BioTek. IC_50_ values were determined by nonlinear regression fitting of the competition curves (GraphPad Prism 6.0 Software). The K_i_ values were calculated as described previously^76^.

### Surface plasmon resonance (SPR) based binding assay

The solution competitive SPR-based assay was performed on Biacore 2000 in a similar way as was already reported^37^. Briefly, N terminal biotin-labelled Bim BH3 peptide (141–166 amino acids) was immobilized on streptavidin (SA) chip giving a density of 800 RU (Response Units). The pre-incubated Mcl-1 protein (20 nM) with tested small-molecule inhibitors for at least 30 min was injected over the surfaces of the chip using assay buffer HBS-P (GE, #BR100368). Response units were measured at 15 s in the dissociation phase, and IC_50_ values were determined by nonlinear least-squares analysis using GraphPad Prism 6.0 software.

### Time-resolved fluorescence energy transfer (TR-FRET) binding assay

TR-FRET was performed using black 384-well plates in an Envision Multilabel plate reader (Perkin Elmer Life Sciences). To each well, a mixture of 10 nM Bid-Dy647 peptide (79–99), 10 nM Mcl-1 protein, and Europium-anti-His antibody were added to a final volume of 25 μl in the assay buffer (20 mM potassium phosphate, pH 7.5; 50 mM NaCl, 1 mM EDTA and 0.05% Pluronic F68). The TR-FRET signals were measured with an Envision Multilabel plate reader after 2 h of incubation. (Ex 340 nM, Em615 nM and Em665 nM) and expressed as FRET signal ratio (F665 nm/F620 nm * 104)

### ^1^H,^15^N HSQC NMR Experiments

NMR studies were performed as previously described^37^. In brief, the ^15^N-labeled Mcl-1 protein was prepared and purified using the same protocol as for unlabelled protein with the exception that the bacteria were grown on M9 minimal media supported with 1 g/L of (^15^NH_4_)_2_SO_4_. Protein samples were prepared in a 20 mM sodium phosphate, 150 mM NaCl and 1 mM DTT solution at pH 7 in 7% D_2_O. The binding mode of compounds has been characterized with a solution of uniformly ^15^N-labeled Mcl-1 (75 μM) in the absence and presence of added compounds (in 1 or 6% final DMSO) with the indicated molar ratio concentrations. The total volume of each sample was 150 μL and samples were prepared in Bruker tubes (Bruker, # Z117724). All Spectra were acquired at 30 °C on a Bruker 600 MHz NMR spectrometer equipped with a cryogenic probe, processed using Bruker TopSpin and rNMR, and were analysed with Sparky. Plots of chemical shift changes were calculated as ((Δ^1^H ppm)2 + (0.2(Δ^15^N ppm))2)0.5 of Mcl-1 amide upon addition of compound. The absence of a bar in a chemical shift plot indicates no chemical shift difference or the presence of a proline or residue that is overlapped or not assigned. The cutoff threshold for determining the significance of the chemical shift was based on one standard deviation above the average of all chemical shifts^77^.

### Bio-Layer Interferometry (BLI) based direct binding assay

Mcl-1 and mutant Mcl-1 R263A proteins were biotinylated using the Thermo EZ-link Sulpho-NHS-LC-biotin biotinylation kit (cat. 21435). Protein and biotin were mixed in a 1:1 molar ratio in PBS on ice for 2 hours. The reaction mixture was dialyzed in PBS buffer to remove excess biotin. BLI experiments were performed using an OctetRED96 instrument from PALL/ForteBio. All experiments were performed at 30 °C using PBS (Gibco, #10010-23), and 0.005% Tween 20 as the assay buffer. Assays were conducted in 96-well, black, microplates (Greiner bio-one, #655209) containing the protein solutions for immobilization, pure assay buffer for dissociation, and serial dilutions of compounds in the presence of 5% DMSO to be tested. During the experiment, sample plates were continuously shaken at 1000 rpm. Biotinylated proteins were immobilized on Super Streptavidin (SSA) biosensors by dipping sensors into plate wells containing protein solutions. Biotinylated blocked Streptavidin sensors were used as control sensors prepared by the protocol provided from the manufacturer. Association (3 Minutes) and dissociation (3 Minutes) cycles of compounds were started by dipping sensors to compound wells with different tested concentrations as well as in pure buffer wells. Buffer-only reference was included in all assays. Raw kinetic data collected were processed with the Data Analysis software provided by the manufacturer using double reference subtraction in which both buffer-only reference and inactive protein reference were subtracted.

### Biotin-Streptavidin Pulldown

2LMP cells, a subclone of MDA-MB-231, were harvested (~15 × 10^6^) and lysed via sonication with CHAPS buffer (10 mM HEPES pH 7.4, 2.5 mM EDTA, 150 mM NaCl, 1% (w/v) CHAPS, pH 7.4). Pre-cleared cell lysates (1 mg/mL) were treated with compounds and then subject to an overnight incubation at 4 °C with biotinylated Noxa peptide (18–43 residues). Protein-peptide complexes were pulled down with agarose beads for 2 hrs. Beads were washed with CHAPS buffer and Mcl-1 protein was eluted by boiling in SDS-PAGE loading dye. Samples were analysed by western blotting with the Mcl-1 antibody (Thermo Scientific #MS-681-P1).

### Cell viability Assays

MEF cells, including wild-type, Bax/Bak double knockout, Bax−/− single knockout, and Bak−/− single knockout were tested and characterized (Supplementary Fig. S3). The cells were cultured in DMEM (Life Technologies), supplemented with 10% fetal bovine serum (FBS) (Thermo Scientific HyClone) and 1% Penicillin/streptomycin solution (Life Technologies). The retrovirally transduced lymphoma cells isolated from Eμ-myc transgenic mice were provided by Ricky W. Johnstone at the University of Melbourne, Melbourne, Australia and cultured as previously described^37,65^.

MEFs were seeded in 24-well plates at 0.5 × 10^5^ cells/well, left to adhere for 5 hours and then treated for 15 hours with increasing concentrations of the compounds. The cells were harvested, washed with phosphate-buffered saline (PBS) and stained with 0.025 mg/ml propidium iodide (MP Biomedicals). The percentage of the propidium iodide positive population was determined by flow cytometry and calculated using WinList 3.0.

The Mcl-1, Bcl-2, and Bcl-xL retroviral transduced lymphoma Eμ-myc cells were seeded in 12-well plates at 0.5 × 10^6^ cells/well. They were treated with different concentrations of tested compounds for 16 hours. The cells were harvested and stained with violet LIVE/DEAD Fixable Dead Cell Stain Kit (Invitrogen) according to manufacturer’s protocol. The percentage of fluorescent positive cells was determined by flow cytometry and calculated using WinList 3.0.

### Data Availability statement

All data generated or analysed during the study are included in this published article (and its Supplementary Information files).

## Electronic supplementary material

Supplementary Material
