# Supplementary material for: Discovery of Mcl-1 inhibitors from integrated high throughput and virtual screening
Source: Sci Rep. 2018 Jul 5;8:10210. doi: 10.1038/s41598-018-27899-9 (PMC6033896; doi:10.1038/s41598-018-27899-9)
Supplement: Supplementary file 1 — Supplementary Material [file 41598_2018_27899_MOESM1_ESM.pdf]

Discovery of Mcl-1 inhibitors from integrated high throughput and virtual screening

Ahmed S.A. Mady,[†,‡,§] Chenzhong Liao,[†,§,1] Naval Bajwa,[†,§,2] Karson J. Kump,[†,¶] Fardokht A. Abulwerdi,[†,‡,3] Katherine L. Lev,[†] Lei Miao,[†] Sierrah M. Grigsby,[†,#] Andrej Perdih,[4] Jeanne A. Stuckey,[△] Yuhong Du,[5] Haian Fu,[5] Zaneta Nikolovska-Coleska[†,‡,#,¶,*]

[†]Department of Pathology and [#]Molecular and Cellular Pathology Graduate Program, University of Michigan Medical School, Ann Arbor, MI, USA; [‡]Interdepartmental Graduate Program in Medicinal Chemistry, College of Pharmacy, University of Michigan; [¶]Program in Chemical Biology, University of Michigan; [4]National Institute of Chemistry, Ljubljana, Slovenia; [△]Life Sciences Institute, University of Michigan; [5]Department of Pharmacology, Emory University, Atlanta, GA, USA

[§]These authors contributed equally.

Current address: [1]School of Medical Engineering, Hefei University of Technology, Hefei, Anhui 230009, China; [2]Pfizer Inc., Lake Forest, IL 60045; [3]Basic Research Laboratory, National Cancer Institute, Frederick, MD 21702

***Corresponding Author**

Phone: +1-734-615-9202; Fax: +1-734-763-8764;

E-mail: zanetan@med.umich.edu

Material and Methods

1. List of compounds purchased from vendors

Compound **1** was purchased from **Sigma-Aldrich** (#S406783).

Compounds **2, 3, 4 and S1, S2** were purchased from **SPECS** (# AN-023/40856766, AN-979/41970861, AF-399/42017468, AN-988/40680316, AG-690/11972094).

Compounds **5, 6, 7, 8** and **S3, S4, S5, S6 S7, S8, S9, S10, S17** were purchased from **Princeton biomolecular research** (# OSSK_137592, OSSK_456180, OSSK_442240, OSSK_461893, OSSK_311111, OSSK-418592, OSSK_523323, OSSK_456099, OSSK_310035, OSSK_316882, OSSK_035875, OSSK_098437, OSSK_399554).

Compounds **9, 10, 11** and **S11**, **S12, S13** were purchased from **Interbioscreen** (# STOCK1S-28802, STOCK5S-07435, STOCK3S-70360, STOCK4S-25927, STOCK4S-63860, STOCK2S-20173).

Compounds **12, 13, 14, 15, 16, 17, 18 and S14, S15, S16** and were purchased from **Enamine** (# T5316685, T0514-7105, T5841620, T0519-0195, T5335131, T0513-6969, T5240340, T0503-1386, T5260297, T0513-7069).

2. Protein purification

His-tagged proteins containing human Mcl-1 (residues 171–323), the isoform 2 construct of the human Bcl-2 [Bcl2-2 construct for protein production starts with the Bcl2 sequence of 1-34 aa, followed by the Bcl-xL sequence of 35-50 aa, and ends with the Bcl-2 sequence of 92-202aa.], Bcl-xL (Human Bcl-xL protein, which has an internal deletion for the 45-85 amino acid residues and a C-terminal truncation for the amino acid residues 212-233), and Bfl-1 (residues 1-151), were expressed from the pHis-TEV vector (a modified pET vector) in E. coli BL21 (DE3) cells. Cells were grown at 37 ºC in 2×YT containing antibiotics to an OD600 density of 1.5, 1 and 0.8 for Mcl-1, Bcl-xL and Bcl-2 respectively. Protein expression was induced by 0.4 mM IPTG at 20 ºC overnight for all proteins except for Bfl-1 which was expressed at 37 ºC for four hours. Cells were lysed in 20 mM HEPES pH 7.5, 200 mM NaCl, 0.1% βME with Leupeptin/Aprotinin (Mcl-1), 25mM Tris pH 8.5, 200mM NaCl (Bcl-2), 50mM Tris pH 7.5, 200 mM NaCl (Bcl-xL) and 50 mM Tris pH 8, 500 mM NaCl, 0.1% βME with Leupeptin/Aprotinin (Bfl-1). All

proteins were purified from the soluble fraction using Ni-NTA resin (QIAGEN), following the manufacturer's instructions. For purification of TEV-cleaved protein, the amino-terminal His tag was cleaved by incubation with TEV protease and the protein was further purified by anion exchange (Source Q) and gel filtration chromatography (Superdex 75, Amersham Biosciences) in 20mM HEPES pH 7.0, 50 mM NaCl (Mcl-1), 25 mM Tris pH 8.5, 150 mM NaCl, 0.1% βME (Bcl-2), 20 mM Tris pH 7.5, 150 mM NaCl, 0.1% βME (Bcl-xL) and 25 mM Tris pH 7.5, 150 mM NaCl, 25% Glycerol, 0.1% βME (Bfl-1). His-tagged Mcl-1 protein was used for TR-FRET assay and HSQC-NMR. His-TEV cleaved Mcl-1, Bcl-2, Bcl-xL, and Bfl-1 were used in fluorescent polarization and surface plasmon resonance binding assays.

Mutant Mcl-1 protein was prepared by the QuikChange site-directed mutagenesis method (Stratagene), where Arg263 was mutated to Ala. The R263A expression plasmid was transformed into Rosetta2 DE3 cells and purified by the same method as the wildtype protein. Cultures were grown to an OD_{600} of 1.5 at 37 °C in Terrific Broth, induced with 0.4 mM IPTG, and expressed overnight at 20 °C. The follow-up purification was performed the same way as was for Mcl-1 wild-type recombinant protein.

Table S1. Docking score of the identified Mcl-1 inhibitors and their fitting into the pharmacophore model.

Compound	Glide SP Docking Score (kcal/mol)	Pharmacophore model fit
**1**	-7.09	Arg263, h4
**2**	-5.23	Arg263, h2, h3
**3**	-5.51	Arg263, h2, h4
**4**	-3.55	Arg263, h2, h4
**5**	-5.43	Arg263, h2, h3
**6**	-5.91	Arg263, h2
**7**	-5.31	Arg263, h2, h3
**8**	-8.11	Arg263, h2, h3
**9**	-6.11	Arg263, h2, h3
**10**	-9.56	Arg263, h2
**11**	-8.83	Arg263, h2, h3
**12**	-3.86	Arg263, h2
**13**	-4.70	Arg263, h2, h3
**14**	-5.67	Arg263, h2
**15**	-3.17	Arg263, h2, h3
**16**	-5.42	Arg263, h2, h4
**17**	-5.20	Arg263, h2
**18**	-9.41	Arg263, h2, h3
**19**	-4.91	Arg263, h2, h3

Table S2. Chemical structures of 17 unconfirmed compounds which didn't show consistent binding results in all biochemical and biophysical assays.

Cpd	Structure	Cpd	Structure	Cpd	Structure
**S1**	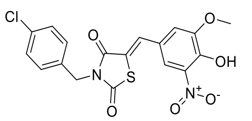	**S7**	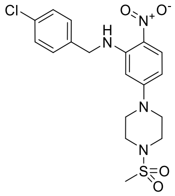	**S13**	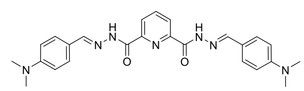
**S2**	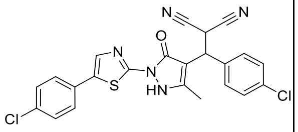	**S8**	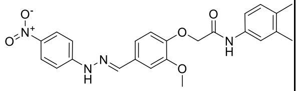	**S14**	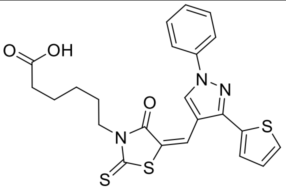
**S3**	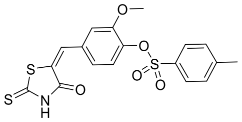	**S9**	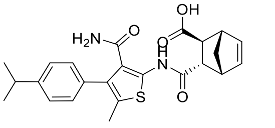	**S15**	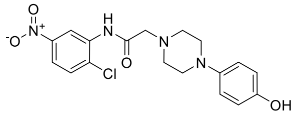
**S4**	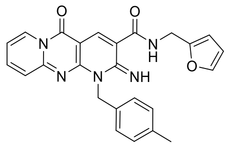	**S10**	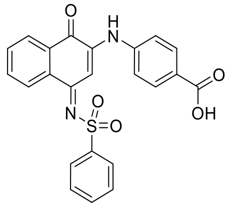	**S16**	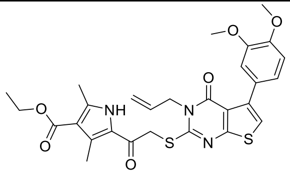
**S5**	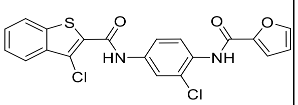	**S11**	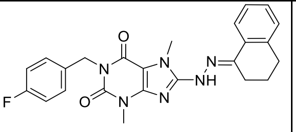	**S17**	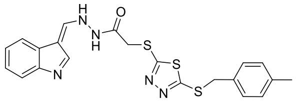
**S6**	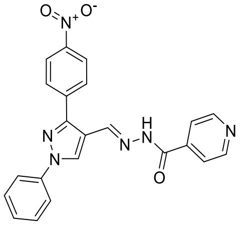	**S12**	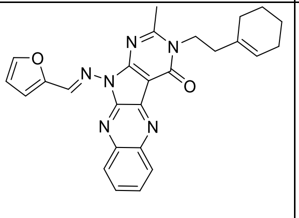		

Table S3. Selectivity profile of validated hit compound 19 and its analogues with improved binding affinity to Mcl-1.

Comp.	Mcl-1		Bfl-1		Bcl-2		Bcl-xL	
	IC_{50} [μM]	K_i [μM]	IC_{50} [μM]	K_i [μM]	IC_{50} [μM]	K_i [μM]	IC_{50} [μM]	K_i [μM]
**19**	13.8±1.4	3.2±0.3	35.6±8.6	4.9±1.2	>100	>25	>100	>20
**39**	5.1±0.5	1.2±0.1	75.9±6.2	10.4±0.9	>100	>25	>100	>20
**42**	1.5±0.3	0.4±0.1	11.4±0.1	1.6±0.1	>100	>25	>100	>20
**44**	1.4±0.2	0.3±0.1	5.1±0.1	0.7±0.1	>100	>25	>100	>20

Figure S1:

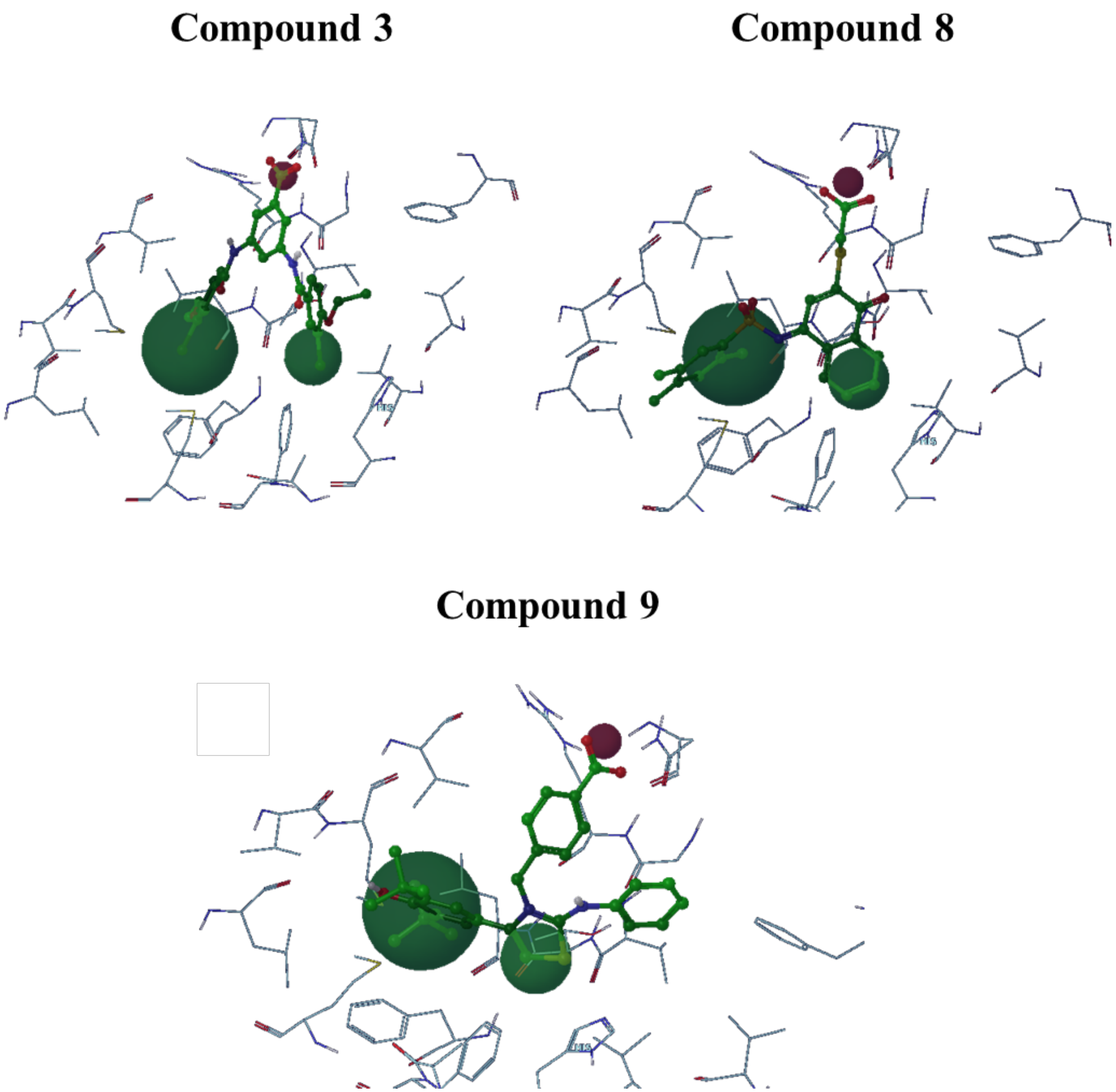

Figure S1: Fitting docking poses of several identified inhibitors to the generated pharmacophore model.

Figure S2:

1

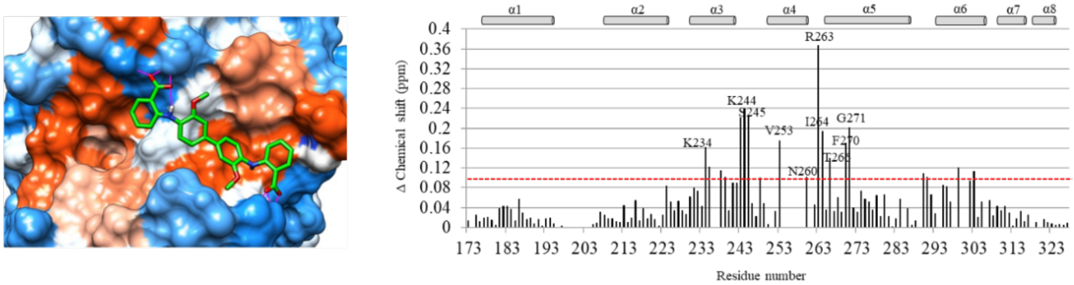
α1 α2 α3 α4 α5 α6 α7 α8
Δ Chemical shift (ppm)
Residue number
K234
K244
S245
V253
N260
R263
I264
G271
F270
L265

2

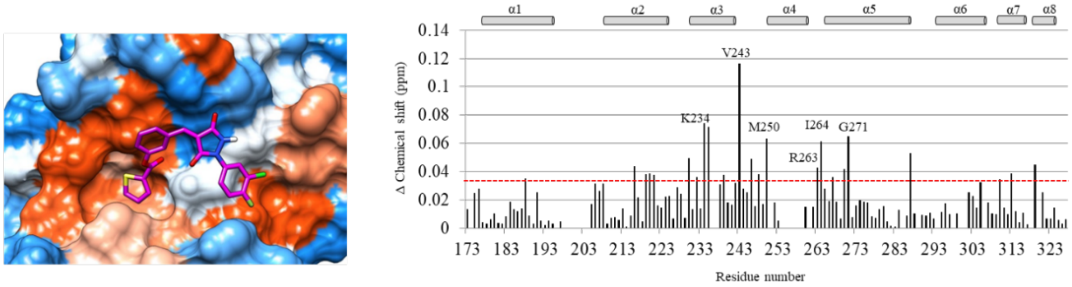
α1 α2 α3 α4 α5 α6 α7 α8
Δ Chemical shift (ppm)
Residue number
V243
K234
M250
I264
G271
R263

3

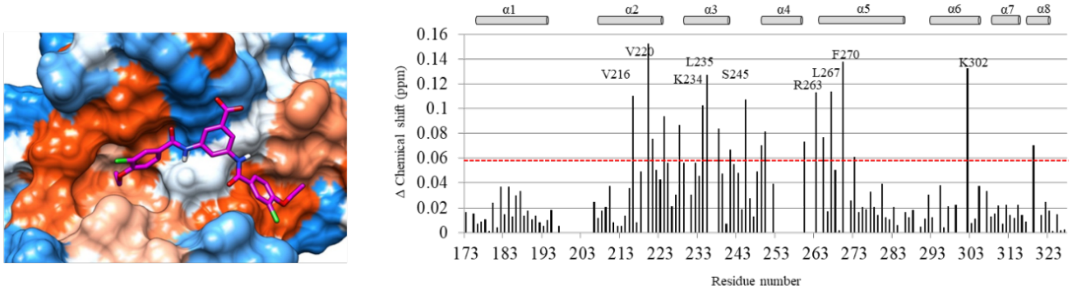
α1 α2 α3 α4 α5 α6 α7 α8
Δ Chemical shift (ppm)
Residue number
V220
V216
L235
K234
S245
F270
L267
R263
K302

4

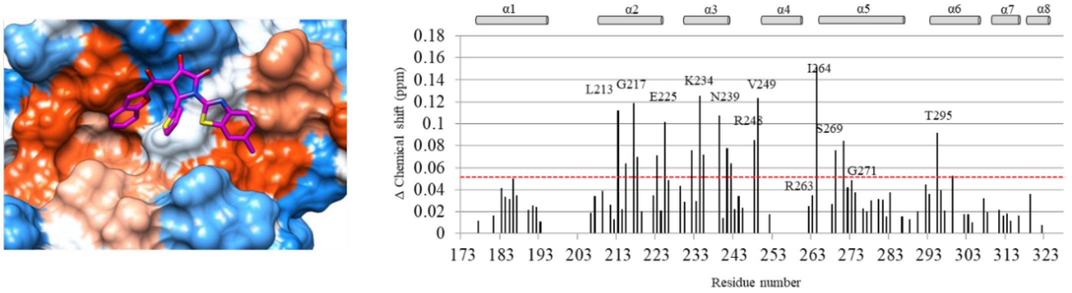
α1 α2 α3 α4 α5 α6 α7 α8
Δ Chemical shift (ppm)
Residue number
L213
G217
E225
K234
N239
V249
R248
I264
S269
G271
R263
T295

6

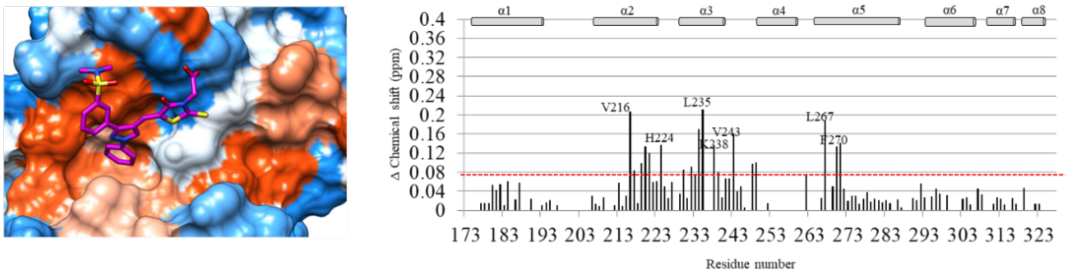
α1 α2 α3 α4 α5 α6 α7 α8
Δ Chemical shift (ppm)
Residue number
V216
H224
L235
K238
V243
L267
F270

7

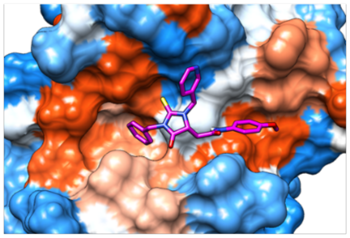

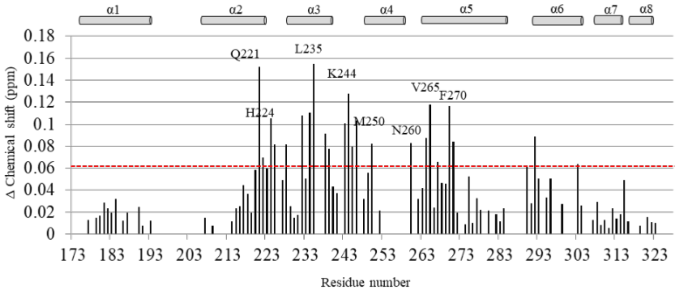
α1 α2 α3 α4 α5 α6 α7 α8
Δ Chemical shift (ppm)
Q221
L235
K244
H224
V265
F270
M250
N260
Residue number

8

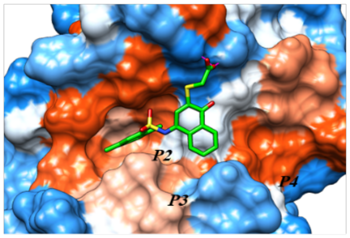
P2
P3
P4

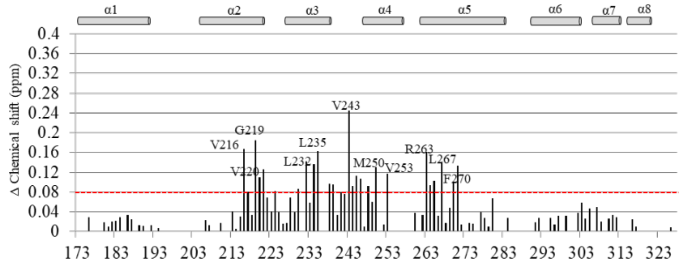
α1 α2 α3 α4 α5 α6 α7 α8
Δ Chemical shift (ppm)
V243
G219
V216
L235
R263
L232
M250
V253
L267
V220
F270
Residue number

9

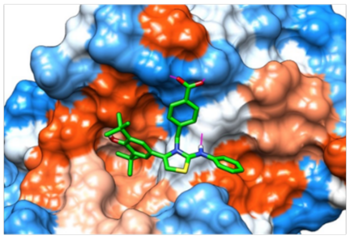

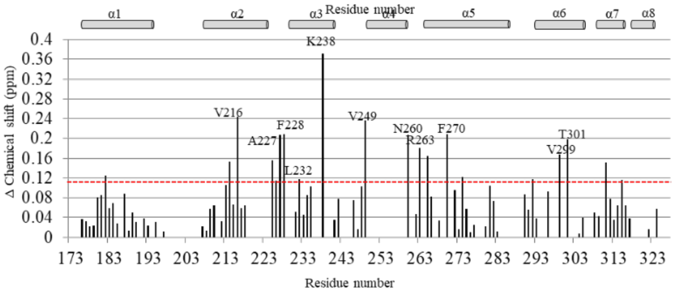
α1 α2 α3 α4 α5 α6 α7 α8
Δ Chemical shift (ppm)
K238
V216
V249
F228
N260
F270
A227
R263
T301
V299
L232
Residue number

10

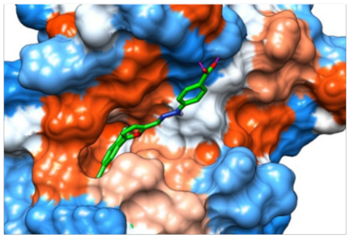

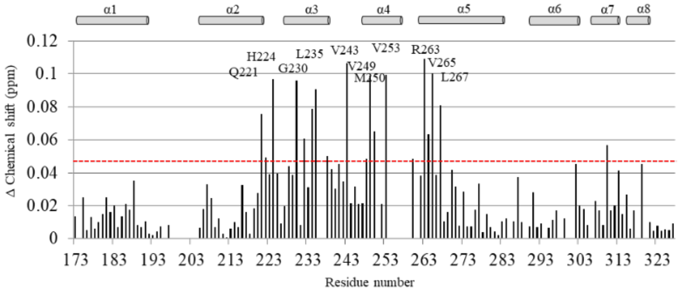
α1 α2 α3 α4 α5 α6 α7 α8
Δ Chemical shift (ppm)
H224
L235
V243
V253
R263
Q221
G230
V249
M250
V265
L267
Residue number

11

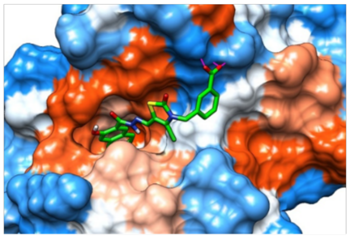

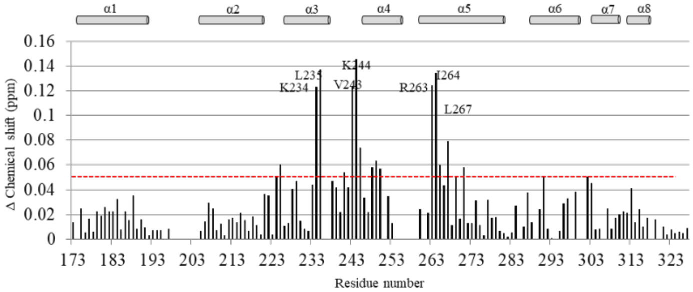
α1 α2 α3 α4 α5 α6 α7 α8
Δ Chemical shift (ppm)
L235
K234
K244
V243
R263
I264
L267
Residue number

12

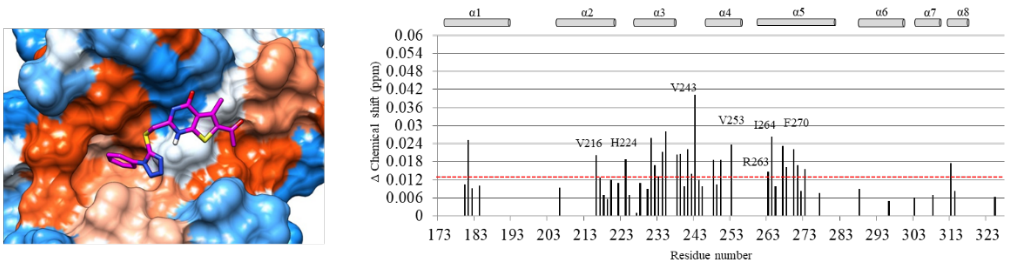

16

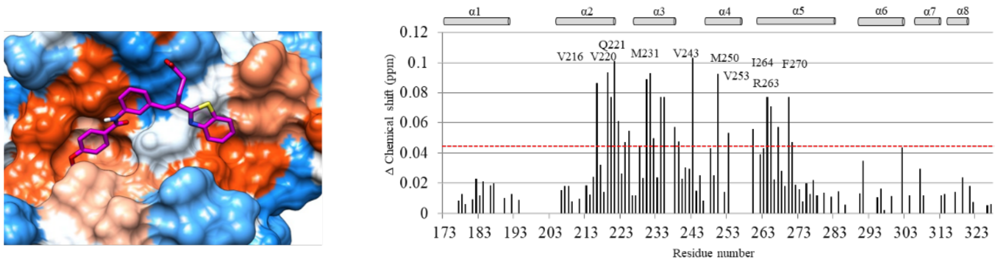

17

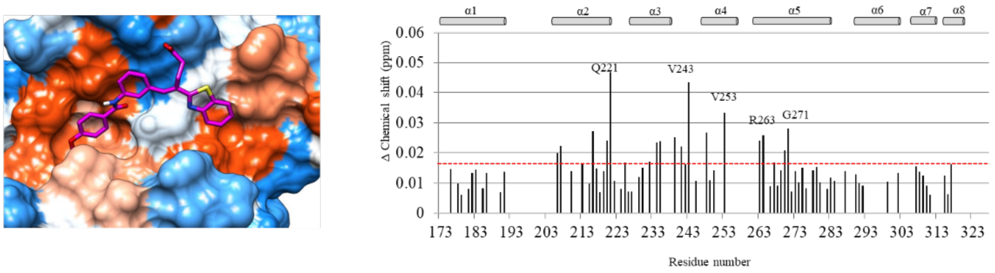

18

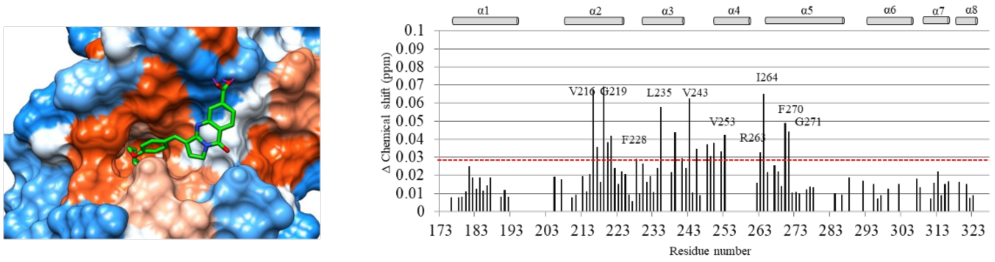

Figure S2. Putative binding mode and the HSQC NMR analysis of the remaining validated hit compounds. Left: Computationally predicted binding poses of the hit compounds in the Mcl-1 binding site using the mNoxa BH3 peptide-bound Mcl-1 crystal structure (PDB: 2NLA). The Mcl-1 protein is colour coded to illustrate hydrophobic (orange) and hydrophilic (blue) surfaces. Right: Plot of the amide chemical shift changes of Mcl-1 in the presence of the corresponding compounds (2 equivalents) as a function of Mcl-1 residue numbers. The red dashed line represents the significance threshold (1 SD above the average chemical shift perturbations).

Figure S3:

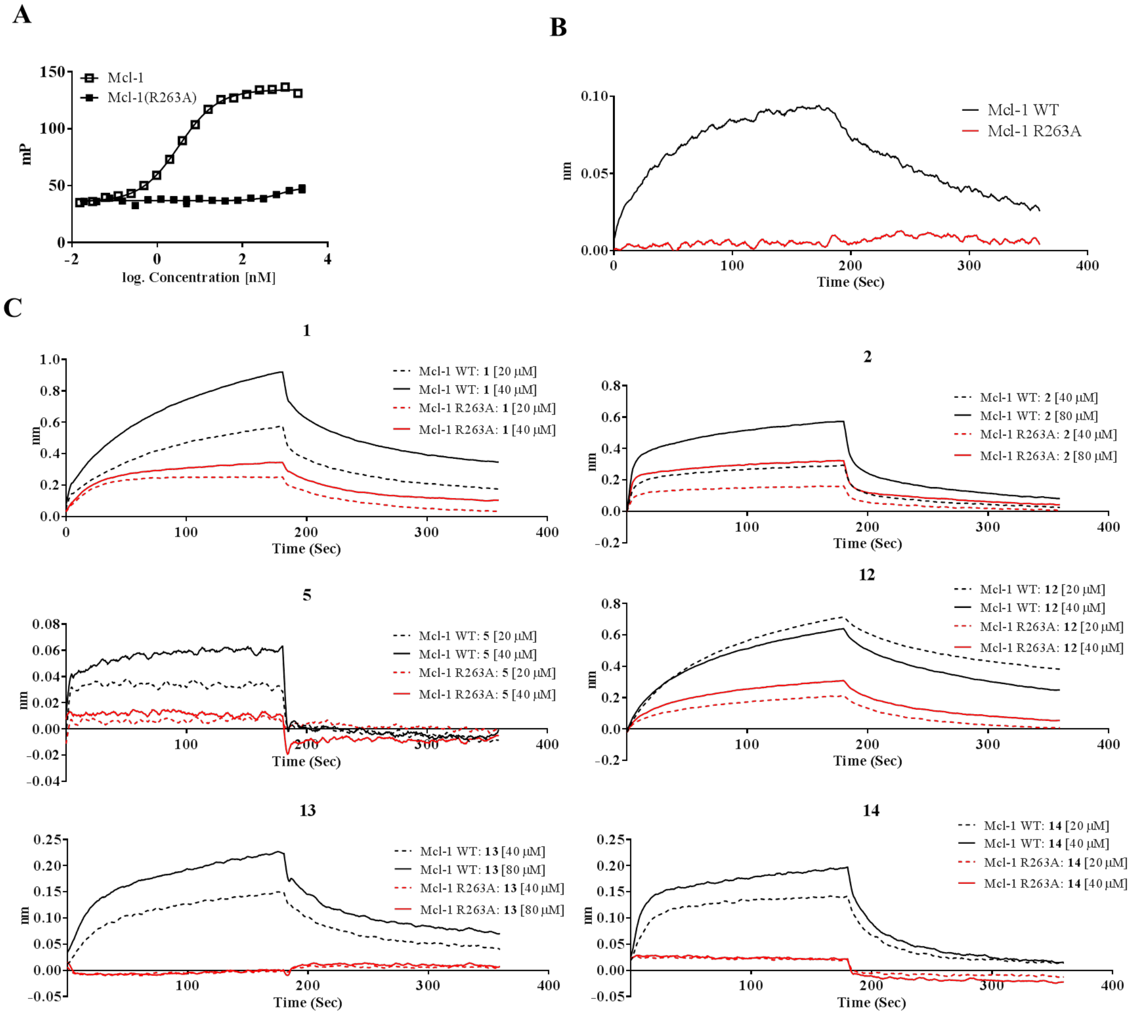

Figure S3: Characterization of the biotin-labelled mutant Mcl-1 R236A and binding of identified hits against this protein versus wild-type Mcl-1: (A) FP based binding assay using Flu-Bid BH3 peptide (2 nM); **(B)** Noxa BH3 peptide (1 μM) binding curves to immobilized wild-type and mutant R263A Mcl-1 proteins using BLI binding assay; **(C)** Several identified hits tested against wild-type and mutant R263A Mcl-1 protein.

Figure S4:

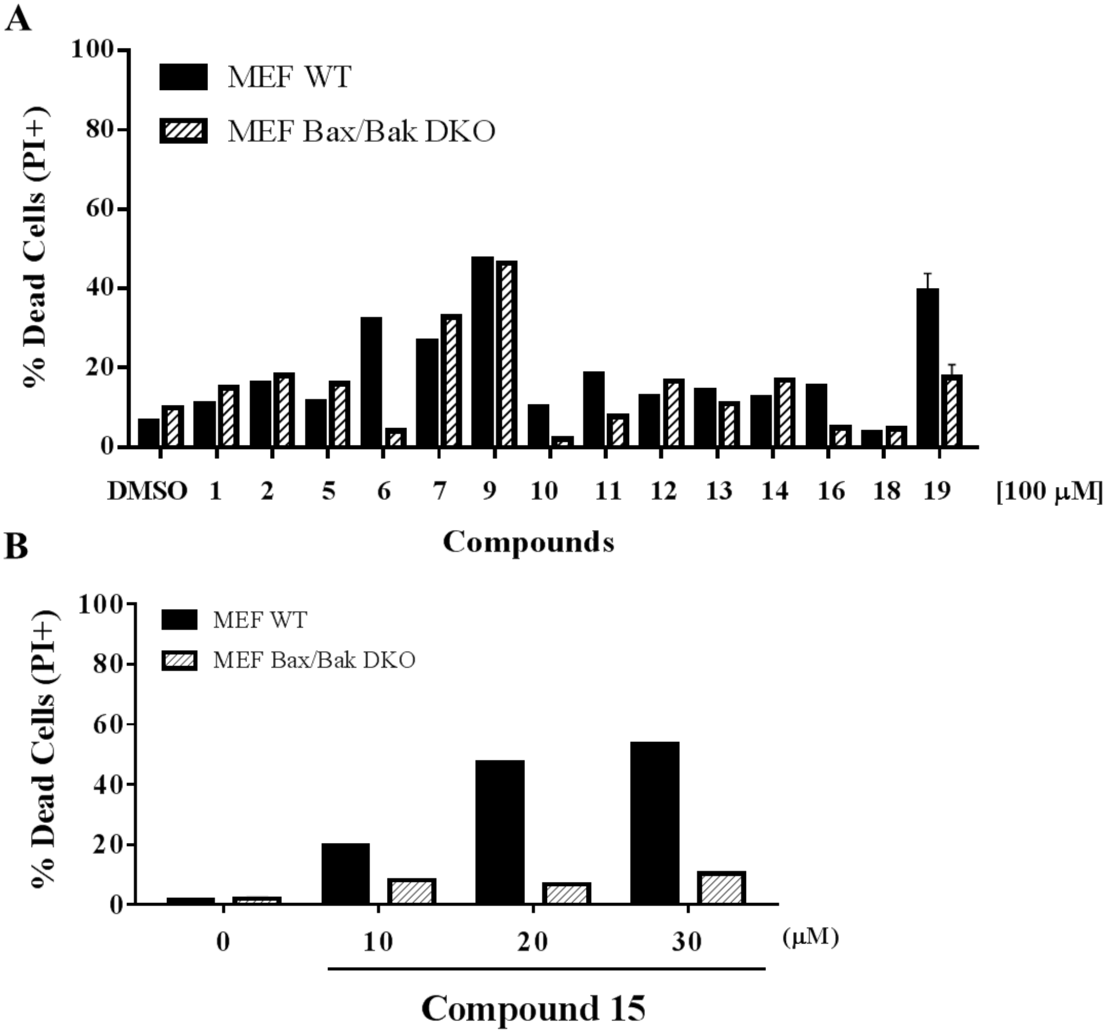

Figure S4: Screening of identified hits for their cellular activity using wild type (WT) and Bax/Bak double knockout (DKO) MEFs. The sensitivity of MEFs WT and DKO to **(A)** 100 μM of identified hits and (**B**) 10, 20, and 30 μM of **15**. Cell viability is determined by propidium iodide staining after 15-hour treatment with compounds.

Figure S5:

A

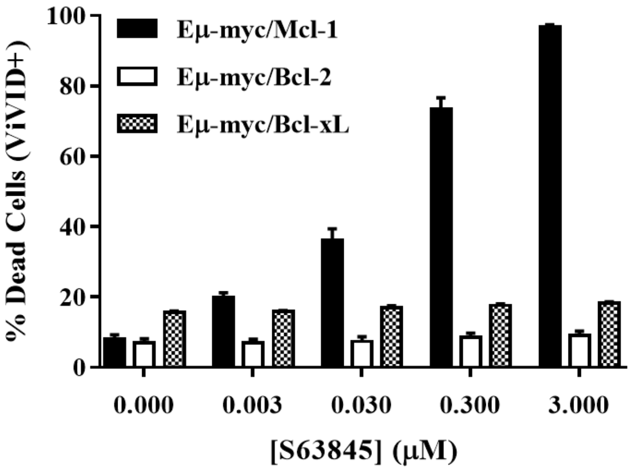

B

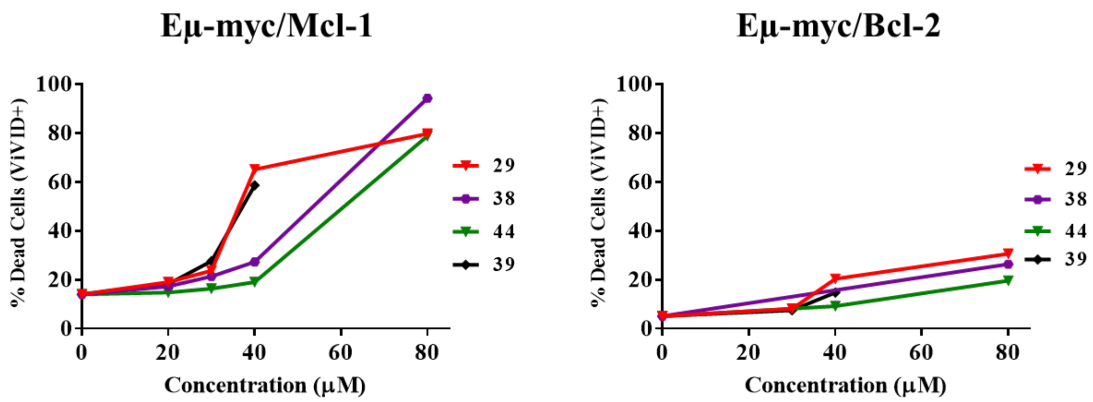

Figure S5. Cellular selectivity of S63845 and developed compounds using Eμ-myc lymphoma cells overexpressing Mcl-1, Bcl-2 and Bcl-xL. (**A**) S63845 is showing selective killing of the Mcl-1 cell line while sparing both Bcl-2 and Bcl-xL overexpressing cell lines. **(B)** Developed Mcl-1 inhibitors **29**, **38**, **44**, and **39** are showing the killing of Mcl-1 expressing cell line while demonstrating selectivity over the Bcl-2 cell line.

Figure S6:

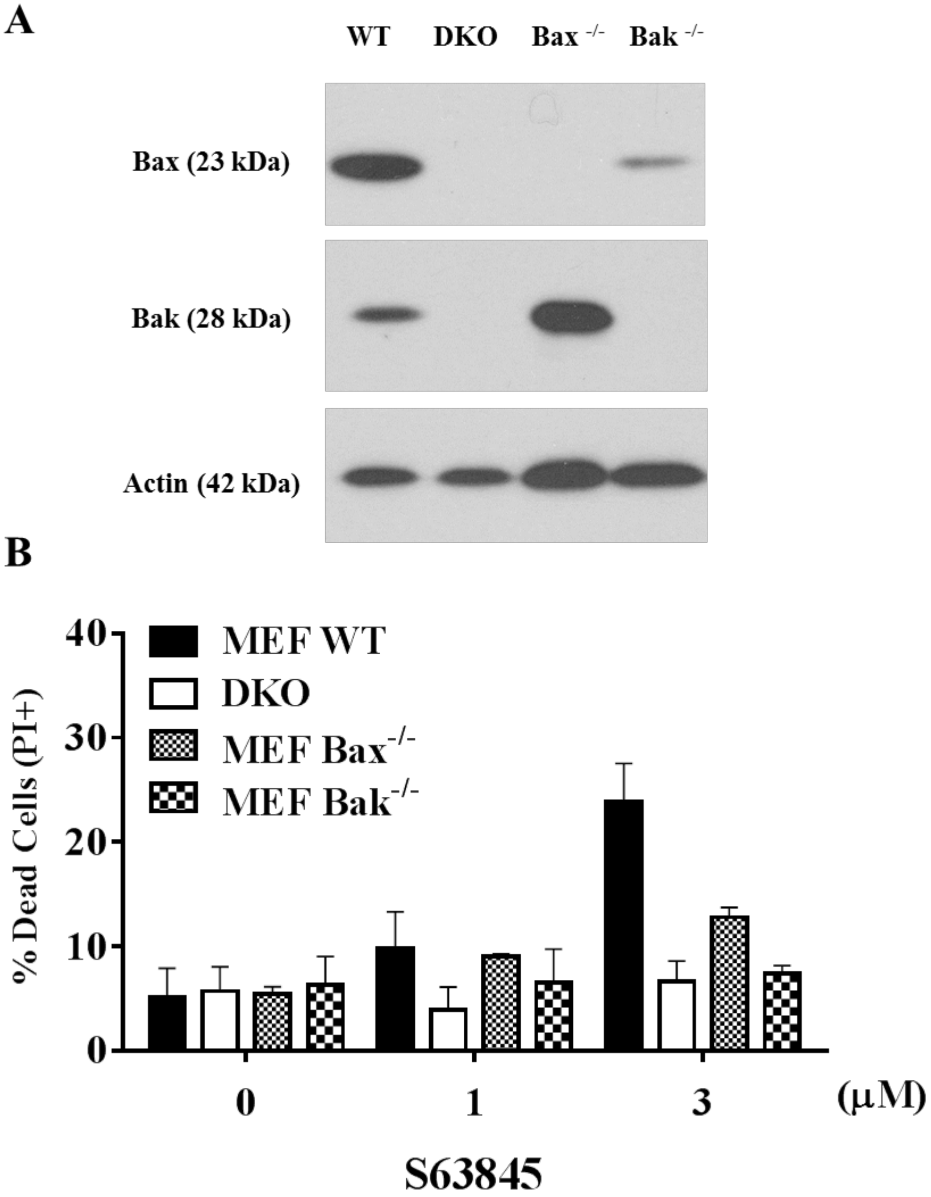

Figure S6. Characterization of MEF cell lines and the Bax/Bak-dependent cell death induction by S63845. (A) Western blot analysis of MEF cell lines, confirming the knockout of Bax and/or Bak. **(B)** S63845 induces cell death in MEF WT but not DKO, $Bax^{-/-}$, or $Bak^{-/-}$.

Full-size blots/gels

Figure 5A

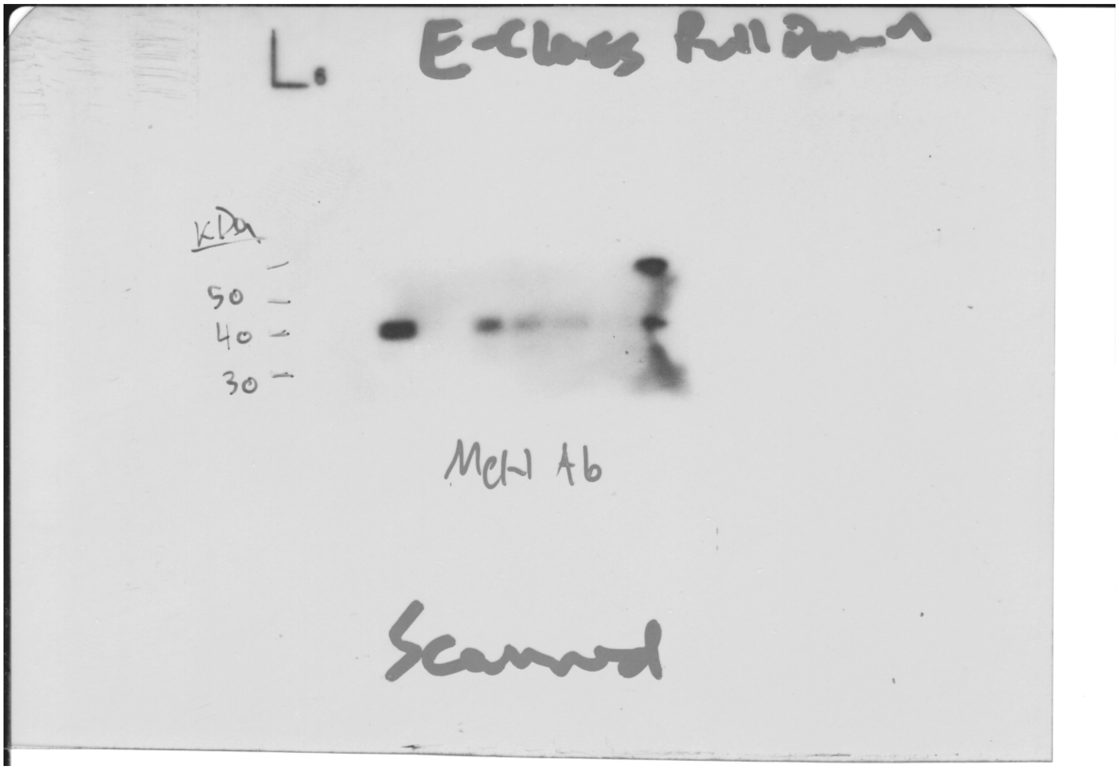

Figure S6:

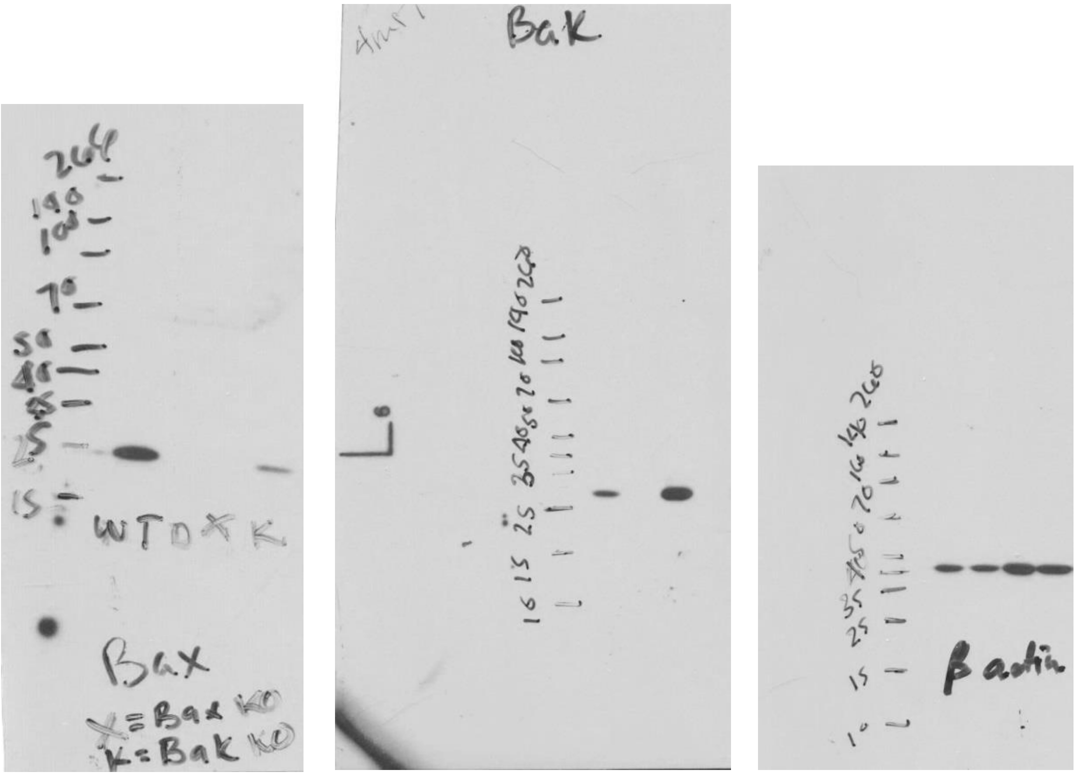

Chemical characterization of synthesized compounds

2-((5,6-di(furan-2-yl)-1,2,4-triazin-3-yl)thio)-N-phenylacetamide (19)

^{1}H NMR (300 MHz, $CDCl_3$) δ 9.24 (s, 1H), 7.69 (ddd, J = 12.0, 1.7, 0.8 Hz, 2H), 7.53 – 7.44 (m, 2H), 7.32 – 7.25 (m, 2H), 7.15 – 7.04 (m, 2H), 6.91 (dd, J = 3.7, 0.8 Hz, 1H), 6.65 (ddd, J = 20.9, 3.6, 1.8 Hz, 2H), 4.05 (s, 2H) ppm.

^{13}C NMR (75 MHz, $CDCl_3$) δ 169.71, 166.68, 147.67, 147.57, 144.41, 144.24, 143.32, 137.82, 128.95, 119.91, 119.74, 113.38, 113.34, 112.36, 35.34 ppm.

HRMS (EI) calcd for C19H14N4O3S [M+Na]+: 401.0684; found, 401.0685.

2-((5,6-di(thiophen-2-yl)-1,2,4-triazin-3-yl)thio)-N-phenylacetamide (25)

^{1}H NMR (300 MHz, $CDCl_3$) δ 9.09 (s, 1H), 7.62 (ddt, *J* = 6.0, 5.0, 0.9 Hz, 2H), 7.56 – 7.41 (m, 4H), 7.29 (td, *J* = 5.7, 5.0, 3.7 Hz, 2H), 7.17 (dd, *J* = 5.1, 3.6 Hz, 1H), 7.14 – 7.00 (m, 2H), 4.07 (s, 2H) ppm.

^{13}C NMR (101 MHz, $CDCl_3$) δ 169.00, 166.31, 149.60, 147.68, 138.10, 137.71, 135.96, 133.84, 133.03, 129.51, 129.39, 128.90, 128.61, 127.78, 124.36, 119.87, 35.21 ppm.

HRMS (EI) calcd for C19H14N4OS3 [M+H]+: 411.0402; found, 411.0401.

2-((5,6-diphenyl-1,2,4-triazin-3-yl)thio)-N-phenylacetamide (26)

^{1}H NMR (300 MHz, $CDCl_3$) δ 9.21 (s, 1H), 7.66 – 7.02 (m, 15H), 4.10 (s, 2H) ppm.

^{13}C NMR (101 MHz, $CDCl_3$) δ 169.71, 166.56, 156.17, 154.65, 137.84, 134.75, 134.62, 131.30, 129.86, 129.74, 129.31, 128.88, 128.69, 128.63, 124.29, 119.77, 35.40 ppm.

HRMS (EI) calcd for C23H18N4OS [M+H]+: 399.1274; found, 399.1276.

2-((5,6-di(pyridin-2-yl)-1,2,4-triazin-3-yl)thio)-N-phenylacetamide (27)

^{1}H NMR (300 MHz, $CDCl_3$) δ 9.26 (s, 1H), 8.39 (tdd, *J* = 4.9, 1.8, 0.9 Hz, 2H), 8.11 (dt, *J* = 7.8, 1.1 Hz, 1H), 8.02 – 7.86 (m, 2H), 7.82 (td, *J* = 7.7, 1.8 Hz, 1H), 7.61 – 7.46 (m, 2H), 7.42 – 7.20 (m, 4H), 7.16 – 7.04 (m, 1H), 4.11 (s, 2H) ppm.

^{13}C NMR (101 MHz, $CDCl_3$) δ 170.55, 166.39, 155.32, 154.10, 154.01, 153.48, 149.01, 148.78, 137.77, 136.98, 136.87, 128.86, 124.95, 124.77, 124.37, 123.82, 123.79, 119.95, 35.41 ppm.

HRMS (EI) calcd for C21H16N6OS [M+H]+: 401.1179; found, 401.1178.

2-((5,6-di(furan-2-yl)-1,2,4-triazin-3-yl)thio)-N-(4-ethylphenyl)acetamide (28)

^{1}H NMR (300 MHz, $CDCl_3$) δ 9.13 (s, 1H), 7.69 (ddd, *J* = 13.1, 1.7, 0.8 Hz, 2H), 7.50 – 7.35 (m, 2H), 7.21 – 7.03 (m, 3H), 6.92 (dd, *J* = 3.7, 0.8 Hz, 1H), 6.65 (ddd, *J* = 20.5, 3.5, 1.8 Hz, 2H), 4.04 (s, 2H), 2.60 (q, *J* = 7.6 Hz, 2H), 1.20 (t, *J* = 7.6 Hz, 3H).

^{13}C NMR (75 MHz, $CDCl_3$) δ 169.72, 166.49, 147.64, 147.60, 144.38, 144.20, 143.28, 140.51, 135.43, 128.27, 120.04, 119.68, 113.36, 113.30, 112.34, 35.28, 28.30, 15.67 ppm.

HRMS (EI) calcd for C21H18N4O3S [M+Na]+: 429.0997; found, 429.0992.

2-((5,6-di(furan-2-yl)-1,2,4-triazin-3-yl)thio)-N-(4-isopropylphenyl)acetamide (29)

^{1}H NMR (300 MHz, $CDCl_3$) δ 9.14 (s, 1H), 7.69 (ddd, *J* = 14.3, 1.6, 0.8 Hz, 2H), 7.51 – 7.36 (m, 2H), 7.25 – 7.03 (m, 3H), 6.91 (dd, *J* = 3.6, 0.8 Hz, 1H), 6.65 (ddd, *J* = 20.0, 3.5, 1.7 Hz, 2H), 4.04 (s, 2H), 2.86 (p, *J* = 6.8 Hz, 1H), 1.21 (d, *J* = 6.9 Hz, 6H).

^{13}C NMR (75 MHz, $CDCl_3$) δ 169.71, 166.52, 147.64, 145.14, 144.38, 144.19, 143.25, 135.49, 126.83, 120.05, 119.69, 113.37, 113.29, 112.34, 35.27, 33.58, 24.01 ppm.

HRMS (EI) calcd for C22H20N4O3S [M+H]+: 421.1329; found, 421.1331.

2-((5,6-di(furan-2-yl)-1,2,4-triazin-3-yl)thio)-N-(4-methoxyphenyl)acetamide (30)

^{1}H NMR (300 MHz, $CDCl_3$) δ 9.09 (s, 1H), 7.68 (ddd, *J* = 10.8, 1.8, 0.8 Hz, 2H), 7.45 – 7.33 (m, 2H), 7.09 (dd, *J* = 3.5, 0.9 Hz, 1H), 6.91 (dd, *J* = 3.7, 0.8 Hz, 1H), 6.86 – 6.75 (m, 2H), 6.64 (ddd, *J* = 21.1, 3.6, 1.7 Hz, 2H), 4.03 (s, 2H), 3.77 (s, 3H).

^{13}C NMR (75 MHz, $CDCl_3$) δ 169.74, 166.43, 156.43, 147.63, 147.60, 144.39, 144.19, 143.26, 130.94, 121.69, 119.67, 114.07, 113.36, 113.30, 112.34, 55.45, 35.20 ppm.

HRMS (EI) calcd for C20H16N4O4S [M+H]+: 409.0965; found, 409.0970.

2-(5,6-di(furan-2-yl)-1,2,4-triazin-3-ylthio)-N-(4-phenoxyphenyl)acetamide (31)

^{1}H NMR (300 MHz, $CDCl_3$) δ 9.23 (s, 1H), 7.70 – 7.65 (m, 2H), 7.45 – 6.89 (m, 11H), 6.68 – 6.59 (m, 2H), 4.03 (s, 2H) ppm.

^{13}C NMR (75 MHz, $CDCl_3$) δ 169.69, 166.59, 157.49, 153.43, 147.70, 147.60, 147.52, 144.46, 144.34, 144.19, 143.29, 133.33, 129.71, 123.00, 121.62, 121.45, 119.75, 119.58, 118.34, 113.34, 112.33, 35.20 ppm.

HRMS (EI) calcd for C25H18N4O4S [M+H]+: 471.1122; found, 471.1116.

2-((5,6-di(furan-2-yl)-1,2,4-triazin-3-yl)thio)-N-(4-(dimethylamino)phenyl)acetamide (32)

^{1}H NMR (300 MHz, $CDCl_3$) δ 8.94 (s, 1H), 7.68 (dt, *J* = 14.6, 4.1 Hz, 2H), 7.37 – 7.21 (m, 2H), 7.09 (d, *J* = 3.5 Hz, 1H), 6.91 (t, *J* = 4.6 Hz, 1H), 6.70 – 6.61 (m, 4H), 4.04 (s, 2H), 2.90 (s, 6H).

^{13}C NMR (101 MHz, $CDCl_3$) δ 169.76, 166.07, 147.98, 147.66, 147.63, 147.49, 144.26, 144.12, 143.10, 127.67, 121.64, 119.49, 113.25, 113.13, 112.89, 112.23, 52.92, 35.17, 8.01 ppm.

HRMS (EI) calcd for C21H19N5O3S [M+H]+: 422.1281; found, 422.1283.

N-(4-aminophenyl)-2-((5,6-di(furan-2-yl)-1,2,4-triazin-3-yl)thio)acetamide (33)

^{1}H NMR (300 MHz, $CDCl_3$) δ 8.95 (s, 1H), 7.75 – 7.58 (m, 2H), 7.29 (s, 1H), 7.27 – 7.22 (m, 1H), 7.14 – 7.04 (m, 1H), 6.91 (t, *J* = 3.3 Hz, 1H), 6.68 (dq, *J* = 3.6, 1.9 Hz, 1H), 6.62 (ddd, *J* = 8.4, 4.4, 2.2 Hz, 3H), 4.03 (s, 2H), 3.55 (bs, 1H), 3.54 (bs, 1H).

^{13}C NMR (101 MHz, $CDCl_3$) δ 206.89, 169.71, 166.17, 147.61, 147.51, 144.30, 144.13, 143.29, 129.15,121.84, 119.53, 116.68, 115.28, 113.25, 113.24, 113.20, 112.25, 35.10 ppm.

HRMS (EI) calcd for C19H15N5O3S [M+H]+: 394.0968; found, 394.0962.

2-((5,6-di(furan-2-yl)-1,2,4-triazin-3-yl)thio)-N-(4-fluorophenyl)acetamide (34)

^{1}H NMR (300 MHz, $CDCl_3$) δ 9.29 (s, 1H), 7.69 (ddd, *J* = 10.8, 1.7, 0.7 Hz, 2H), 7.45 (ddt, *J* = 9.2, 6.7, 3.3 Hz, 2H), 7.11 (dd, *J* = 3.4, 0.8 Hz, 1H), 7.06 – 6.81 (m, 3H), 6.66 (ddd, *J* = 20.8, 3.6, 1.8 Hz, 2H), 4.04 (s, 2H) ppm.

^{13}C NMR (75 MHz, $CDCl_3$) δ 169.68, 166.70, 160.97, 157.74, 147.69, 147.53, 144.46, 144.24, 143.33, 133.88, 133.85, 121.72, 121.62, 119.79, 115.71, 115.41, 113.41, 113.39, 112.37, 35.24 ppm.

HRMS (EI) calcd for C19H13FN4O3S [M+H]+: 397.0765; found, 397.0772.

N-([1,1'-biphenyl]-4-yl)-2-((5,6-di(furan-2-yl)-1,2,4-triazin-3-yl)thio)acetamide (35)

^{1}H NMR (300 MHz, DMSO) δ 10.51 (s, 1H), 7.97 (ddd, *J* = 10.7, 1.7, 0.8 Hz, 2H), 7.74 – 7.68 (m, 2H), 7.67 – 7.62 (m, 4H), 7.49 – 7.40 (m, 2H), 7.37 – 7.28 (m, 1H), 7.06 (dd, *J* = 3.4, 0.9 Hz, 1H), 6.99 (dd, *J* = 3.6, 0.8 Hz, 1H), 6.75 (ddd, *J* = 6.9, 3.6, 1.8 Hz, 2H), 4.32 (s, 2H) ppm.

^{13}C NMR (75 MHz, DMSO) δ 170.03, 166.11, 148.95, 147.92, 147.90, 145.50, 144.08, 142.99, 140.08, 138.90, 135.53, 129.36, 127.52, 127.46, 126.71, 119.97, 119.36, 113.82, 113.23, 112.69, 35.88 ppm.

HRMS (EI) calcd for C25H18N4O3S [M+H]+: 455.1172; found, 455.1168.

N-(4-benzylphenyl)-2-((5,6-di(furan-2-yl)-1,2,4-triazin-3-yl)thio)acetamide (36)

^{1}H NMR (400 MHz, $CDCl_3$) δ 9.15 (s, 1H), 7.79 – 7.55 (m, 2H), 7.47 – 6.96 (m, 10H), 6.85 (dd, *J* = 3.7, 0.5 Hz, 1H), 6.60 (ddd, *J* = 29.3, 3.6, 1.8 Hz, 2H), 3.99 (s, 2H), 3.88 (s, 2H) ppm.

^{13}C NMR (101 MHz, $CDCl_3$) δ 169.65, 166.56, 147.63, 147.53, 147.49, 144.35, 144.15, 143.22, 141.02, 137.23, 135.86, 129.37, 128.79, 128.40, 126.02, 120.04, 119.70, 113.35, 113.29, 112.32, 41.28, 35.25 ppm.

HRMS (EI) calcd for C26H20N4O3S [M+H]+: 469.1329; found, 469.1324.

2-((5,6-di(furan-2-yl)-1,2,4-triazin-3-yl)thio)-N-(3-ethylphenyl)acetamide 37)

^{1}H NMR (300 MHz, $CDCl_3$) δ 9.17 (s, 1H), 7.67 (ddd, *J* = 12.8, 1.8, 0.8 Hz, 2H), 7.40 – 7.27 (m, 2H), 7.26 – 7.13 (m, 1H), 7.08 (dd, *J* = 3.5, 0.8 Hz, 1H), 7.02 – 6.81 (m, 2H), 6.64 (ddd, *J* = 19.6, 3.5, 1.8 Hz, 2H), 4.04 (s, 2H), 2.58 (q, *J* = 7.6 Hz, 2H), 1.18 (t, *J* = 7.6 Hz, 3H).

^{13}C NMR (101 MHz, $CDCl_3$) δ 169.63, 166.51, 147.59, 147.55, 145.16, 144.33, 144.11, 143.22, 137.76, 128.79, 123.95, 119.60, 119.35, 117.23, 113.31, 113.24, 112.28, 35.33, 28.76, 15.40 ppm.

HRMS (EI) calcd for C21H18N4O3S [M+H]+: 407.1172; found, 407.1173.

2-((5,6-di(furan-2-yl)-1,2,4-triazin-3-yl)thio)-N-(3-isopropylphenyl)acetamide (38)

^{1}H NMR (300 MHz, $CDCl_3$) δ 9.11 (s, 1H), 7.78 – 7.52 (m, 2H), 7.39 – 7.14 (m, 3H), 7.14 – 7.02 (m, 1H), 7.02 – 6.79 (m, 2H), 6.65 (ddt, J = 20.8, 4.9, 2.3 Hz, 2H), 4.06 (s, 2H), 2.83 (tp, J = 13.1, 6.8 Hz, 1H), 1.21 (s, 3H), 1.17 (s, 3H).
^{13}C NMR (101 MHz, $CDCl_3$) δ 169.61, 166.52, 149.82, 147.58, 144.34, 144.09, 143.19, 137.74, 128.80, 122.50, 119.61, 118.02, 117.44, 113.33, 113.24, 112.29, 35.33, 34.02, 23.82 ppm.
HRMS (EI) calcd for C22H20N4O3S [M+H]+: 421.1329; found, 421.1331.

2-((5,6-di(furan-2-yl)-1,2,4-triazin-3-yl)thio)-N-(3-(trifluoromethyl)phenyl)acetamide (39)

^{1}H NMR (300 MHz, $CDCl_3$) δ 9.55 (s, 1H), 7.82 – 7.62 (m, 4H), 7.48 – 7.31 (m, 2H), 7.12 (d, J = 3.5 Hz, 1H), 6.90 (d, J = 3.7 Hz, 1H), 6.66 (ddt, J = 19.1, 2.9, 1.6 Hz, 2H), 4.05 (s, 2H) ppm.

^{13}C NMR (101 MHz, $CDCl_3$) δ 169.58, 167.01, 147.67, 147.41, 147.40, 144.43, 144.20, 143.37, 138.35, 131.37, 131.05, 129.45, 122.92, 122.38, 120.82, 119.86, 116.48, 113.43, 113.42, 112.35, 35.29 ppm.

HRMS (EI) calcd for C20H13F3N4O3S [M+H]+: 447.0733; found, 447.0735.

N-([1,1'-biphenyl]-3-yl)-2-((5,6-di(furan-2-yl)-1,2,4-triazin-3-yl)thio)acetamide (40)

^{1}H NMR (300 MHz, $CDCl_3$) δ 9.32 (s, 1H), 7.73 – 7.63 (m, 3H), 7.52 (tq, J = 7.8, 3.5, 2.6 Hz, 3H), 7.47 – 7.30 (m, 5H), 7.10 (t, J = 3.4 Hz, 1H), 6.92 (t, J = 3.4 Hz, 1H), 6.64 (dddt, J = 23.6, 5.1, 3.2, 1.7 Hz, 2H), 4.07 (s, 2H) ppm.

^{13}C NMR (101 MHz, $CDCl_3$) δ 169.60, 166.71, 147.61, 147.56, 147.55, 144.35, 144.12, 143.24, 141.95, 140.50, 138.25, 129.30, 128.64, 127.41, 127.09, 123.13, 119.67, 118.78, 118.62, 113.34, 113.29, 112.30, 35.37 ppm.

HRMS (EI) calcd for C25H18N4O3S [M+H]+: 455.1172; found, 455.1172.

N-([1,1'-biphenyl]-2-yl)-2-((5,6-di(furan-2-yl)-1,2,4-triazin-3-yl)thio)acetamide (41)

^{1}H NMR (300 MHz, $CDCl_3$) δ 8.99 (s, 1H), 8.08 (d, *J* = 8.0 Hz, 1H), 7.62 (dd, *J* = 32.3, 1.8 Hz, 2H), 7.38 (td, *J* = 8.5, 7.5, 2.5 Hz, 1H), 7.24 – 7.04 (m, 5H), 7.00 (t, *J* = 7.4 Hz, 2H), 6.96 – 6.86 (m, 1H), 6.79 (d, *J* = 3.6 Hz, 1H), 6.69 (t, *J* = 2.9 Hz, 1H), 6.56 (td, *J* = 2.7, 1.3 Hz, 1H), 3.94 (s, 2H) ppm.

^{13}C NMR (75 MHz, $CDCl_3$) δ 168.99, 166.95, 147.80, 147.52, 147.44, 144.31, 143.68, 142.89, 138.04, 134.35, 134.06, 130.14, 128.76, 128.16, 127.34, 125.10, 123.52, 119.36, 113.23, 113.06, 112.35, 34.94 ppm.

HRMS (EI) calcd for C25H18N4O3S [M+Na]+: 477.0997; found, 477.0990.

5,6-di(furan-2-yl)-3-((3-phenylpropyl)thio)-1,2,4-triazine (42)

^{1}H NMR (300 MHz, $CDCl_3$) δ 7.64 (dd, *J* = 11.2, 1.7 Hz, 2H), 7.43 – 7.14 (m, 5H), 7.04 (d, *J* = 3.4 Hz, 1H), 6.83 (d, *J* = 3.6 Hz, 1H), 6.61 (ddd, *J* = 23.9, 3.4, 1.6 Hz, 2H), 3.36 (t, *J* = 7.2 Hz, 2H), 2.86 (t, *J* = 7.5 Hz, 2H), 2.32 – 2.10 (m, 2H) ppm.

^{13}C NMR (101 MHz, $CDCl_3$) δ 170.79, 148.29, 148.14, 146.89, 143.96, 143.94, 142.56, 141.15, 128.49, 128.40, 125.96, 118.18, 112.74, 112.48, 112.05, 34.85, 30.70, 30.21 ppm.

HRMS (EI) calcd for C20H17N3O2S [M+H]+: 364.1114; found, 364.1110.

5,6-di(furan-2-yl)-3-(phenethylthio)-1,2,4-triazine (43)

^{1}H NMR (300 MHz, $CDCl_3$) δ 7.57 (ddd, *J* = 13.4, 1.7, 0.8 Hz, 2H), 7.33 – 7.08 (m, 5H), 7.02 – 6.88 (m, 1H), 6.81 – 6.68 (m, 1H), 6.62 – 6.39 (m, 2H), 3.56 – 3.38 (m, 2H), 3.16 – 2.96 (m, 2H) ppm.

^{13}C NMR (101 MHz, $CDCl_3$) δ 170.68, 148.24, 148.12, 146.91, 144.03, 143.98, 142.65, 140.00, 128.73, 128.48, 126.50, 118.24, 112.80, 112.55, 112.08, 35.72, 32.17 ppm.

HRMS (EI) calcd for C19H15N3O2S [M+H]+: 350.0958; found, 350.0954.

5,6-di(furan-2-yl)-3-((4-phenylbutyl)thio)-1,2,4-triazine (44)

^{1}H NMR (300 MHz, $CDCl_3$) δ 7.64 (ddd, *J* = 10.2, 1.6, 0.8 Hz, 2H), 7.36 – 7.14 (m, 5H), 7.04 (dd, *J* = 3.4, 0.8 Hz, 1H), 6.85 (dd, *J* = 3.6, 0.8 Hz, 1H), 6.61 (ddd, *J* = 24.7, 3.4, 1.7 Hz, 2H), 3.37 (t, *J* = 6.7 Hz, 2H), 2.70 (t, *J* = 7.1 Hz, 2H), 1.98 – 1.79 (m, 4H).

^{13}C NMR (101 MHz, $CDCl_3$) δ 170.89, 148.32, 148.15, 146.88, 143.96, 143.94, 142.54, 142.05, 128.37, 128.29, 125.75, 118.15, 112.74, 112.47, 112.06, 35.41, 30.65, 30.57, 28.74 ppm.

HRMS (EI) calcd for C21H19N3O2S [M+H]+: 378.1271; found, 378.1273.

2,3-di(furan-2-yl)-N-phenylquinoxaline-6-carboxamide (45)

^{1}H NMR (300 MHz, $CDCl_3$) δ 8.56 (s, 1H), 8.36 – 8.17 (m, 2H), 8.09 (s, 1H), 7.78 – 7.62 (m, 4H), 7.50 – 7.38 (m, 2H), 7.26 – 7.17 (m, 1H), 6.79 (ddd, *J* = 15.6, 3.5, 0.8 Hz, 2H), 6.62 (dt, *J* = 3.4, 1.6 Hz, 2H) ppm.

^{13}C NMR (101 MHz, $CDCl_3$) δ 164.43, 150.43, 150.37, 144.71, 144.59, 143.71, 143.36, 141.85, 139.65, 137.71, 136.27, 129.78, 129.16, 128.85, 127.25, 124.84, 120.12, 114.06, 113.73, 112.13, 112.06, 109.99 ppm.

HRMS (EI) calcd for C23H15N3O3 [M+H]+: 382.1186; found, 382.1185.

2,3-di(furan-2-yl)-N-(p-tolyl)quinoxaline-6-carboxamide (46)

^{1}H NMR (300 MHz, $CDCl_3$) δ 8.50 (s, 1H), 8.32 – 8.05 (m, 3H), 7.65 (dt, *J* = 1.7, 0.9 Hz, 2H), 7.62 – 7.53 (m, 2H), 7.24 – 7.14 (m, 2H), 6.75 (ddd, *J* = 13.9, 3.5, 0.8 Hz, 2H), 6.60 (dt, *J* = 3.5, 1.8 Hz, 2H), 2.36 (s, 3H).

^{13}C NMR (101 MHz, $CDCl_3$) δ 164.43, 150.43, 150.37, 144.72, 144.59, 143.71, 143.36, 141.85, 139.65, 137.71, 136.27, 129.78, 129.16, 128.85, 127.25, 124.84, 120.12, 114.06, 113.73, 112.13, 112.06 ppm.

HRMS (EI) calcd for C24H17N3O3 [M+H]+: 396.1343; found, 396.1344.
